# Mussel-inspired conductive Ti_2_C-cryogel promotes functional maturation of cardiomyocytes and enhances repair of myocardial infarction

**DOI:** 10.7150/thno.38876

**Published:** 2020-01-12

**Authors:** Genlan Ye, Zubiao Wen, Feng Wen, Xiaoping Song, Leyu Wang, Chuangkun Li, Yutong He, Sugandha Prakash, Xiaozhong Qiu

**Affiliations:** 1 Guangdong Provincial Key Laboratory of Construction and Detection in Tissue Engineering, School of Basic Medical Science, Biomaterials Research Center, School of Biomedical Engineering, Southern Medical University, Guangdong, Guangzhou 510515, China; 2 College of Chemistry and Chemical Engineering, Jiangxi Normal University, Nanchang 330022, China; 3 Guangzhou Regenerative Medicine and Health Guangdong Laboratory, Guangzhou 510005, China

**Keywords:** MXene Ti_2_C, dopamine, conductive cryogels, vasculation, myocardial infarction

## Abstract

**Rationale**: Researches on conductive engineering cardiac patch (ECP) for myocardial infarction (MI) treatment have achieved some progress in the animal while the availability of traditional conductive materials in ECP is still limited because of their controversial cytotoxicity. Here we aim to introduce a novel hydrophilic biocompatible conductive material: MXene Ti_2_C and mussel-inspired dopamine into PEGDA-GelMA cryogel to construct a bio-functional ECP of which the property closes to natural heart for the repair of MI.

**Method**: MXene Ti_2_C was etched from MAX Ti_2_AlC, then uniformly dispersed into the prepolymer composed with dopamine-N′, N′-methylene-bisacrylamide, methacrylate-gelatin, and poly (ethylene glycol) diacrylate by simple water bath sonication. The resilient conductive Ti_2_C-cryogel was fabricated by chemical cryogelation. The conductive ECP was evaluated *in vitro* and transplanted to the MI rat model for MI treatment.

**Results**: *In vitro*, the 3D vessels-shape framework was observed in Ti_2_C-8-cryogel which was seeded with rats aortic endothelial cells. When the Ti_2_C-cryogels were cocultured with CMs, remarkably aligned sarcomere and the primitive intercalated disc between the mature CMs were formed on day 7. The as-prepared Ti_2_C-8-cryogel ECP also demonstrated rapid calcium transients and synchronous tissue-like beating. When transplanted into the infarcted heart of the MI rat model, the Ti_2_C-8-cryogel ECP could improve the cardiac function, reduce the infarct size, and inhibit the inflammatory response. Obvious vasculation especially newly formed arteriole was also found.

**Conclusion**: A novel conductive Ti_2_C-embedded cardiac patch with suitable conductivity and the mechanical property was developed and could be served as an ideal candidate for MI repair.

## Introduction

Myocardial infarction (MI), as a cause of heart dysfunction and heart failure, is still one of the major threats for human health worldwide 1-3. MI-induced heart dysfunctions mainly manifested as weakened electric signal propagation and heart contraction, which triggered by cardiomyocytes (CMs) necrosis, fibrous scar tissue formation, and damaged packed tight-joint cytoarchitecture 4, 5. Traditional therapies for MI, such as thrombolytic therapy, heart bypass surgery, and heart transplantation, are unsatisfying yet due to the limited regeneration ability of CMs and the lack of heart graft sources 6, 7. Therefore, the engineered cardiac patches (ECPs) aiming at constructing functional cardiac tissue, had been considered as a promising strategy for treating MI 8-10.

The introduction of bio-functional conductive materials into the ECPs has been proved to be an effective approach to improve cardiac function after MI 11. These conductive biomaterials include conductive polymers (such as polyaniline 12, polythiophene 13 and polypyrrole 14), carbon nanomaterials (such as graphene 15 and carbon nanotube 16) and metal-based compounds (such as gold nanoparticles 17). It has been demonstrated that conductive materials could promote the maturation of electrical stimuli-responsive cells followed by enhancing the communication between cells 18. Conductive ECPs, as a substitute for infarction tissue, can bridge electrical signals of healthy myocardium across the scar obstacle and activate cardiomyocytes within the scar 19, 20. However, the bioavailability of the above mentioned conductive materials is still limited because of their controversial cytotoxicity and low solubility. The development of some other safe conductive materials with favorable conductivity, good hydrophilicity, and excellent biocompatibility is still a key issue.

MXenes, as a class of two-dimensional inorganic compounds with excellent conductive layered structures, had come into the researcher's sight 21-23. To date, the MXenes were still mainly applied in the energy storage area because of their excellent electrical performance 24-26. Recently, titanium carbide (Ti_2_C), as one kind of MXenes, had been used to fabricate biosensors owned to its enzyme immobilization characteristic 27 and tumor-killing therapy in that it could lead to oxidative stress-induced cancer cell damage 28. Similar to the graphene, Ti_2_C was an emerging two-dimensional conductive material with a layered structure 29. This 2D material could be formed by MAX Ti_2_AlC etching and exist in the form of three to seven expanded separated Ti-C layers, which could facilitate an untrammeled electron shift between layers and therefore displayed high electronic conductivity 30. Ti_2_C flakes have a highly conductive transition metal (Ti) carbide core and a functional surface with active chemical groups (such as -O and -OH) 31. These surface terminations render Ti_2_C hydrophilic. Unlike other conductive materials with low solubility, easy precipitation and aggregation in biological media or polymer solutions 32, the hydrophilicity of Ti_2_C enables it to be uniformly distributed in these similar solutions. Also as one of the inorganic compounds with chemically stable structure, Ti_2_C had been proved well biocompatibility 33. Considering its good conductivity, hydrophilicity, and biocompatibility, we intend to extend the application of Ti_2_C from energy area to biological area. We supposed that Ti_2_C could be used as a candidate material for the construction of functional conductive ECPs.

Besides of conductive materials, the suitable scaffold is also very important for ECP construction. To date, electrospinning fibers 34, 35, decellularized scaffold 36, 37, and hydrogel 38, 39 have been widely utilized to develop ECPs. Among these, the hydrogel is an ideal scaffold due to its versatility of fabrication, excellent biocompatibility, ease of operation, and appropriate mechanical property. For MI treatment, the flexible hydrogel can match the contraction of the heart when fixed to the infarcted region. Dopamine, derived from the mussel foot protein, has been proved its strong adhesion and easy crosslinking property 40. We previously have also produced a mussel-inspired cryogel derived ECP using dopamine-N′, N′-methylene-bisacrylamide (DOPA-MBA), methacrylate-gelatin (MA-G) and poly (ethylene glycol) diacrylate (PEGDA) 41. Conjugated by DOPA-MBA, the cryogel could obtain a strongly adhesive performance and has a good retention ability for cardiac cells. In order to improve the conductive property, we introduced the Ti_2_C into this mussel inspired cryogel to form Ti_2_C-cryogel. Because of its stiff characteristic 42, Ti_2_C can endow the cryogel with stronger mechanical strength and better toughness than the pure cryogel. We found that a cryogel with proper Ti_2_C proportion had a suitable mechanical property and an appropriate conductivity to match the natural myocardium, whose conductivity is about 0.1 S/m and elastic modulus is about 0.03-0.5MPa 43, 44. RAEC could form a 3D tube-like shape when cultured in the Ti_2_C-cryogel for 3 days. Also, when cultured with primary CMs, Ti_2_C-cryogel showed no obvious toxicity to cell growth and promote its functionalization* in vitro*. After transplantation in MI rats, the Ti_2_C-cryogel derived ECPs could significantly improve heart function in MI rats.

## Materials and Methods

### Materials

Gelatin, dopamine, N′, N′-methylene-bisacrylamide (MBA), Poly (ethylene glycol) diacrylate (PEGDA, Mw = 700) tetramethylethylenediamine (TEMED) were all purchased from Sigma (St Louis, USA). Ti_2_AlC has been purchased from 11 technology Co. LTD (Jilin, China). The Live/Dead cell staining kit was from Molecular Probes (Life Technologies). The Cell Counting Kit-8 (CCK-8) was purchased from Dojindo Molecular Technologies (Japan). The primary antibodies of α-actinin, connexin 43 (CX-43), and von Willebrand Factor (vWF) were acquired from Abcam. F4/80 antibody was purchased from Ebioscience (USA). The alpha-smooth muscle actin (α-SMA) was ordered from Boster Biological Technology (Wuhan, China).

### Preparation of DOPA-MBA cross-linker

DOPA-MBA cross-linker was prepared according to the previous report 40. In briefly, 500 mg of MBA (3 mmol) was dissolved in deionized water/ethanol (v/v = 4:3, pH = 6) solution to reach 70.1 mg/ml final concentration. And then, 475 mg dopamine was added into the solution under nitrogen protection. This mixture was stirred at 45 °C for 3 days in darkness and then the DOPA-MBA cross-linker was obtained. The DOPA-MBA cross-linker solution was lyophilized and stored at -20 °C.

### Preparation of MA-G

Gelatin with Double Bond (MA-G) was also prepared according to previous reports 45. In briefly, 1g gelatin and 0.5 ml methacrylic anhydride (MA) were dissolved in 10 ml PBS (pH = 7.4) at 50 °C separately. After being strongly stirred for 1 hour, the reaction was then stopped by adding another 10 ml PBS. After being dialyzed against deionized water and lyophilized, the MA-G was then obtained.

### Synthesis of Ti_2_C and MATi_2_C

Ti_2_C was prepared according to the previous studies 46. MXA Ti_2_ALC powder was etched by a 10% HF aqueous solution for 12 h at room temperature. Then the HF-treated powder was dried in vacuum at 60 °C for 24 h to get the Ti_2_C powder. For the preparation of MATi_2_C, 20 mg Ti_2_C powder was dispersed into 10 ml deionized water, then 0.5ml methacrylic acid was dissolved in this solution at 50 °C and stirred overnight. Finally, the mixture was dialyzed for 3 days and lyophilized, the MATi_2_C powder was obtained.

### Characterization of Ti_2_C nanoparticles

The size and zeta potential distributions of Ti_2_C particles were detected using dynamic light scattering (DLS) (Zetasizer Nano-Zs, Malvern Instruments, UK). The molecular structure of the Ti_2_C solid powder was analyzed by X-ray diffraction (XRD). A high-resolution Empyrean diffractometer (PANalytical) equipped with the CuKα1 X-ray source was used and operated at 40 kV. The 2θ scans were recorded in angles ranging from 5° to 80° (2θ) in a continuous scan mode. The morphology of the Ti_2_C nanoparticles was observed under Transmission Electron Microscopy (TEM) (JSM-2010HR, Japan) at an acceleration voltage of 200 kV.

### Preparation of the Ti_2_C-cryogel

The Ti_2_C-cryogel was synthesized according to the procedures as follows: 46 µl MA-G (6.67 mg) solution, 46 μl PEGDA (3.33 mg) solution, and 4 μl DOPA-MBA crosslinker (0.8 mg) solution were uniformly mixing at 50 °C to prepare cryogel mixture. Then, the Ti_2_C solid powder with different mass (0, 0.4, 0.8 or 1.6 mg), was dispersed into 100 μl deionized water and then added them to the cryogel mixture with water bath sonication for 20 mins to get different prepolymer solution. Finally, 3 μl 10% APS and 1 μl TEMED were added into prepolymer solution and put the final mixture into -20 °C freezer overnight to form different cryogels (Ti_2_C-free cryogel: cryogel without Ti_2_C nanoparticles; Ti_2_C-2-cryogel: Ti_2_C-cryogel with 2 mg/ml Ti_2_C final concentration; Ti_2_C-4-cryogel: Ti_2_C-cryogel with 4 mg/ml Ti_2_C final concentration; Ti_2_C-8-cryogel: Ti_2_C-cryogel with 8 mg/ml Ti_2_C final concentration). To observe the dispersion of Ti_2_C in the prepolymer, the prepolymer solutions of different concentrations of Ti_2_C without APS and TEMED were rest for 72 hours and the graphene oxide prepolymer solution was prepared as a control. As for the MATi_2_C-cryogel preparation, 2 mg/ml, 4 mg/ml and 8 mg/ml MATi_2_C were introduced and the method is similar to the Ti_2_C-cryogel preparation.

### Characterization of the Ti_2_C-cryogels

The micro-structures of the cryogels were observed using Scanning Electron Microscopy (SEM, S-3000N, Hitachi, Japan) and the Titanium distribution on the cryogel was further detected via energy-dispersive X-ray spectroscopy (SEM-EDX, Hitachi, Japan). The conductivity of the Ti_2_C-cryogels was measured using a Digital Four-Point Probe (Suzhou Jingge Electronic Co. LTD, China). For the mechanical properties of different cryogels, all samples were synthesized with 4~6 mm height and 10 mm diameter. The cycling compressive test was executed up to 60% sample deformation at a compressive speed of 10 mm/min for 100 cycles by LS1 materials testing system (AMETEK, America). The stress-strain curves and modulus of different cryogels were also measured in single continued compressive at the same speed.

### Neonatal rat cardiomyocytes isolation and culture

Neonatal rat cardiomyocytes were isolated from 1-3 day old Sprague-Dawley rat hearts as described previously 47. All the procedures were accepted by the Institution of Animal Care (IACUC) at the Southern Medical University Animal Ethics Committee. Briefly, the rat hearts were quickly harvested and dissociated into a cell suspension by 0.25% Trypsin (Sigma) and 0.1% collagenase type II (Sigma). The cardiomyocytes were obtained by pre-plated for 2 hours to remove the fibroblasts. Then harvested cardiomyocytes were seeded in the cryogels (7.5×10^5^/cm^3^) or the glass slide (2×10^5^ cells/cm^2^) and cultured in high-glucose DMEM medium (GIBCO) supplemented with 10% fetal bovine serum (FBS, GIBCO), 100 U/ml penicillin, and 100 μg/ml streptomycin. The cells were cultured in an incubator at 37 ^o^C with 5% CO_2_.

### Biocompatibility evaluation for Ti_2_C nanoparticles and the ROS detection of CMs treated with Ti_2_C nanoparticles

The biocompatibility of Ti_2_C nanoparticles was tested using live/dead staining. After CMs were cultured on glass slides for 1 day, the cells were treated with different concentrations of Ti_2_C nanoparticles (25 μg/ml, 100 μg/ml and 250 μg/ml). At the indicated time, CMs were washed three times with PBS and stained with the live/dead working solution at 37 °C for 30 min in the dark. The images of the dyed samples were captured with a fluorescence microscope (Olympus, BX53, Japan). Cell viabilities were defined as the ratio of living cells. The number of cells was counted at six independent sites of each sample (three samples for each cryogels).

TEM was used for analyzing the CMs' cellular uptake for Ti_2_C nanoparticles. After treated with different concentrations of Ti_2_C nanoparticles for 3 days, the CMs were washed with PBS 3 times to remove the isolated Ti_2_C particles. The CMs were fixed with 2.5% glutaraldehyde and 1.0% osmium tetroxide, then dehydrated with graded ethanol solutions and embedded in Eponate 812 for the ultrathin section preparation. The micrographs were observed under a TEM (Hitachi H-7500).

The ROS production in CMs treated with Ti_2_C nanoparticle was detected with the ROS fluorescence probe-DHE according to the manufacturer's instructions. Then the probe-labeled samples were stained with F-actin FITC and DAPI. The fluorescence images were captured under a fluorescence microscope. The ROS positive area was quantified using Image J software.

### Biocompatibility evaluation for Ti_2_C-cryogels

The viability of CMs cultured on the cryogels was detected by Live/Dead cell staining and CCK-8 assay. CMs on scaffolds were rinsed with PBS three times, followed by incubating in staining solution at 37 °C for 30 min in the dark. Photos concerning stained samples were obtained applying a laser scanning confocal microscope (LSM 880 with Airyscan). The quantifications of cell viability were the same as above. The CCK-8 assay was performed as follows. Briefly, the CMs were seeded in different cryogels in a 96-well culture plate, six duplicate wells for each group. At day 3 and day 7 of culture, the culture medium was replaced with 100 μl working solution (CCK-8 solution/serum-free medium: 10 μl /90 μl). Then after the samples were incubated for 2 hours, the working solutions of all wells were transferred into a new 96-well culture plate for absorbance detection. The OD value was measured at 450 nm using a microplate reader.

### Immunofluorescence analysis for cardiomyocyte on the Ti_2_C-cryogels

On day 3 or day 7 of culture, CMs on different cryogels were fixed with 4% paraformaldehyde (PF) for 1 hour followed by permeabilized with 0.2% Triton X-100 in PBS for 15 min. Then samples were blocked with 2% Bovine Serum Albumin (BSA) at room temperature for 2 hours. Afterward, cells were incubated with primary antibodies, mouse anti-sarcomeric alpha-actinin (1:200) and rabbit anti-CX43 (1:200) at 4 °C overnight. After washed three times with PBS, cells were incubated with secondary antibodies for 2 hours at room temperature (Alexa Fluor 488 donkey anti-mouse IgG (H&L, 1:500) for sarcomeric alpha-actinin and Alexa Fluor 568 donkey anti-rabbit IgG (H&L, 1:500) for CX43). And finally, samples were stained with 4′, 6-diamidino-2-phenylindole (DAPI) for 1 hour and imaged with confocal microscopy (LSM 880 with Airyscan). The CMs α-actinin and CX43 positive area were quantified using Image J software. At least 5 fields of each sample were quantified.

### Western blot analysis

After culturing for 7 days, myocardial samples were frozen in liquid nitrogen, and the total proteins were extracted using RIPA protein extraction solution. The harvested proteins were separated on 10% SDS-PAGE and transferred onto polyvinylidene fluoride (PVDF) membranes. Then the membranes were blocked with 5% skim milk (w/v) in TBS-T buffer (10 mM/L Tris·HCl, pH 7.5, 500 mM/L NaCl, 0.05% Tween 20) for 2 h at room temperature and then incubated with primary antibody against α-actinin, CX-43 or GAPDH at 4 °C overnight. Membranes were then washed 3 times with TBST (10 min each time) and incubated with horseradish peroxidase-linked secondary antibody for 2 h and exposed using chemiluminescence Super ECL Detection Reagent (Shang Hai Yeasen Biological Technology, China). GAPDH was used as an internal control. The experiments were performed for three replicates and the data were analyzed with Image J software.

### Morphology and ultrastructure observation

The morphologies of CMs on different cryogels were observed under SEM (S-3000N). After culturing for 7 days, CMs on cryogels were washed with PBS and fixed with 2.5% of glutaraldehyde overnight. The fixed cells were then dehydrated with graded ethanol (50%, 70%, 90%, and 100%) and freeze-dried. Finally, cells were stained with dioxin and lead citrate. As for the TEM analysis, the samples were washed three times with PBS and fixed with 2.5% glutaraldehyde and 1.0% osmium tetroxide. Next, washed in PBS at 4 °C overnight, then the samples were dehydrated with graded ethanol solutions and embedded with Eponate 812. All samples were sliced into ultrathin sections (about 50 nm), and the micrographs were observed under a TEM (Hitachi H-7500).

### Calcium transients assay

To assess Ca^2+^ transient within CMs on different cryogels, calcium indicator assay kit was utilized. At day 3 after culture, all samples were stained with Fluo-4 AM reagent (a working solution of 100 μg Fluo-4 Am in 100 μl Pluronic F127) for 45 min at 37 °C according to the manufacturer's instructions. After being removed from the indicator solution, the samples with calcium current fluorescence were detected using a fluorescence microscope (Olympus, BX53, Japan). Fluorescence (F) during cell contractions was normalized to the background intensity (F_0_) and plotted over time using Image J software. Three samples for each group were quantified. The contraction behaviors of the Ti_2_C-cryogel ECPs were recorded with a video (Panasonic, HC-X900M, Japan) under microscopy. The videos were digitized at a rate of 25 frames per second, and the beating signals were analyzed and recorded by Image J software.

### Observation and the qPCR analysis for vasculation in the Ti_2_C cryogel *in vitro*

Rat aortic endothelial cells (RAECs) were isolated from SD rats based on the previous works 49. Briefly, the rats' vessels were cut into 2_*_2 mm pieces and placed them on the Matrigel-coated plates cultured with endothelial cells growth medium, a DMEM supplement with 15% FBS, 10 U/ml of heparin, and 75 g/ml. After 3 days of culture, the remained vessel pieces were removed and the RAECs were harvested from the plates and seeded on the different cryogels for 3 days. Then the cells on the cryogels were fixed with 4% paraformaldehyde for the fluorescence staining (n=3) or lysis with Trizol Reagent (Ambion, Carlsbad CA, USA) for the qPCR analysis (n=3). The fixed samples were then incubated with F-actin for 30 min and then snapped by confocal microscopy. The RNA extracted from RAECs was reverse transcribed to cDNA with the Thermo First cDNA Synthesis Kit (Toyobo Co. Ltd., Japan). qPCR was run on a StepOne PLUS system (Applied Biosystems, USA) with SYBR Green Master Mix kit (TaKaRa, Japan) to analyze the expression of the vasculation related gene eNOS and VEGF. GAPDH was used as an internal reference gene. The sequences of PCR primers (forward and backward, 5' to 3') were as follows:

VEGF, 5'-CGGGCCTCTGAAACCATGAA-3' and 5'-GCTTTCTGCTCCCCTTCTGT-3'; eNOS, 5'-GCCCCCAGAACTCTTCACTC' and 5'-CCGGGTGTCTAGATCCATGC -3'.

### Implantation of ECPs into rat MI model

Animal experiments were carried out in accordance with the Regulations for the Administration of Affairs Concerning Experimental Animals (China) and approved by the Southern Medical University Animal Ethics Committee. Male SD rats (7-8 weeks, weight 250 ± 20 g) were divided into the sham group, MI group, Ti_2_C-free cryogel ECP group, Ti_2_C-4-cryogel ECP group, and Ti_2_C-8-cryogel ECP group. MI model was performed as previously described 49, briefly, all rats were anesthetized with isoflurane, performed ventilation and then achieved thoracotomy and ligation operation of the left anterior descending artery (LAD). For the sham group, the only thoracotomy for rats was performed. After 7 days of LAD ligation, the heart functions of rats were evaluated by echocardiography. The MI rats with less than 30% fractional short-ending (FS) value were selected for the next transplantation experiments. Before the ECPs transplantation, CMs were labeled with DiI (3 μg/mL) for cell tracking and then seeded in different cryogels (2 cm diameter and 0.5 cm thickness) for 7 days. Then the various ECPs were transplanted onto the epicardium in the infarction area then and the edge of ECP was fixed with 8-0 suture. For the sham and MI groups, second thoracotomy but not ECPs implantation were performed for rats.

### Cardiac function analysis

The left heart function of all animal groups was evaluated by Vevo2100 echocardiography (Vevo2100, Visual Sonics). Four weeks after patch transplantation, the rats were anesthetized, and echocardiography was performed. M-mode tracings and short-axis views were recorded with an M250 transducer. The cardiac functional parameters, including the left ventricular internal diameter at end-diastole (LVIDd), left ventricular internal diameter at end-systole (LVIDs), left ventricular ejection fraction (EF) and left ventricular shortening fraction (FS) were measured.

### Histological assay

Four weeks after transplantation, all rats were euthanized. then the heart was cut into 3 parts from the apex level to auricular appendix level. Each part was cut about 0.4 cm transverse slices and fixed in 4% paraformaldehyde. Then the slices were dehydrated with 30% sucrose and frozen embedded in O.C.T. for the section. Afterward, 8 μm sections were cut via a Leica CM1950 cryostat. Then Masson trichrome staining was performed according to the manufacturer's instructions. Based on the Masson trichrome staining images, the infarct areas were defined as the ratio of the inner circumference of the fibrous area (blue) to the entire inner circumference in the LV. The wall thickness of the infarct area was also measured with Image J software. The immunostaining for cardiac sections was performed according to the method *in vitro*. Briefly cardiac sections were washed with to remove the O.C.T. then permeabilized with 1% Triton X-100 and blocked with 2% Bovine Serum Albumin (BSA). Subsequently, for cardiac marker detection, cardiac sections were incubated with the primary antibodies of mouse anti-sarcomeric alpha-actinin (1:200) and rabbit anti-CX-43 (1:200); for vessels marker detection, cardiac sections were incubated with mouse anti-α smooth muscle actin (α-SMA) (1:200) and rabbit anti-vWF (1:200); and for inflammation marker detection, cardiac sections were incubated with rabbit anti-F4/80 (diluted concentration: 1:200), mouse anti-CD68 (diluted concentration: 1:50), and rabbit anti-CD163 (diluted concentration: 1:500). Then, all cardiac sections were incubated with corresponding secondary antibody and nuclei were stained with DAPI (Sigma). The images were taken using a fluorescence microscope (Olympus, BX53, Japan). The qPCR analysis was used for evaluating the expression of the vasculation related gene eNOS and VEGF in the infarcted tissue.

### Distribution of the Ti_2_C nanoparticles* in vivo*

The distribution of the Ti_2_C was evaluated by detecting the content of the titanium in the heart (attach with the ECP), lung, liver, and kidney with an inductively coupled plasma-mass spectroscopy (ICP-MS, Thermo Fisher iCAP) according to the previous study. On day 2 and day 28 of Ti_2_C-8-cryogel ECP transplantation, rats (n=3) were euthanized and their hearts, lungs, livers, and kidneys were harvested and then powdered in liquid nitrogen and further lyophilized. Then lyophilized powder from each organ was digested in 5ml aqua fortis and diluted 10 times with deionized water for the ICP-MS detection. The detection was carried out under the condition of 1550W RF power, 0.8 ml/min auxiliary gas flow, 14 L/min cooling gas flow.

### Statistical analysis

All results were analyzed with the SPSS22.0 and GraphPad prism 5 software. The data were expressed as means ± standard deviations (SD). Statistical analyses were performed using one-way analysis of variance (ANOVA). Tukey HSD post hoc testing was used as the post hoc correction to compare multiple groups. To compare the differences between the two groups, two-tailed unpaired Student's t-tests were used. Differences were considered significant at *p* < 0.05.

## Results and Discussion

### Fabrication and characterization of Ti_2_C conductive cryogel

**Scheme 1:**
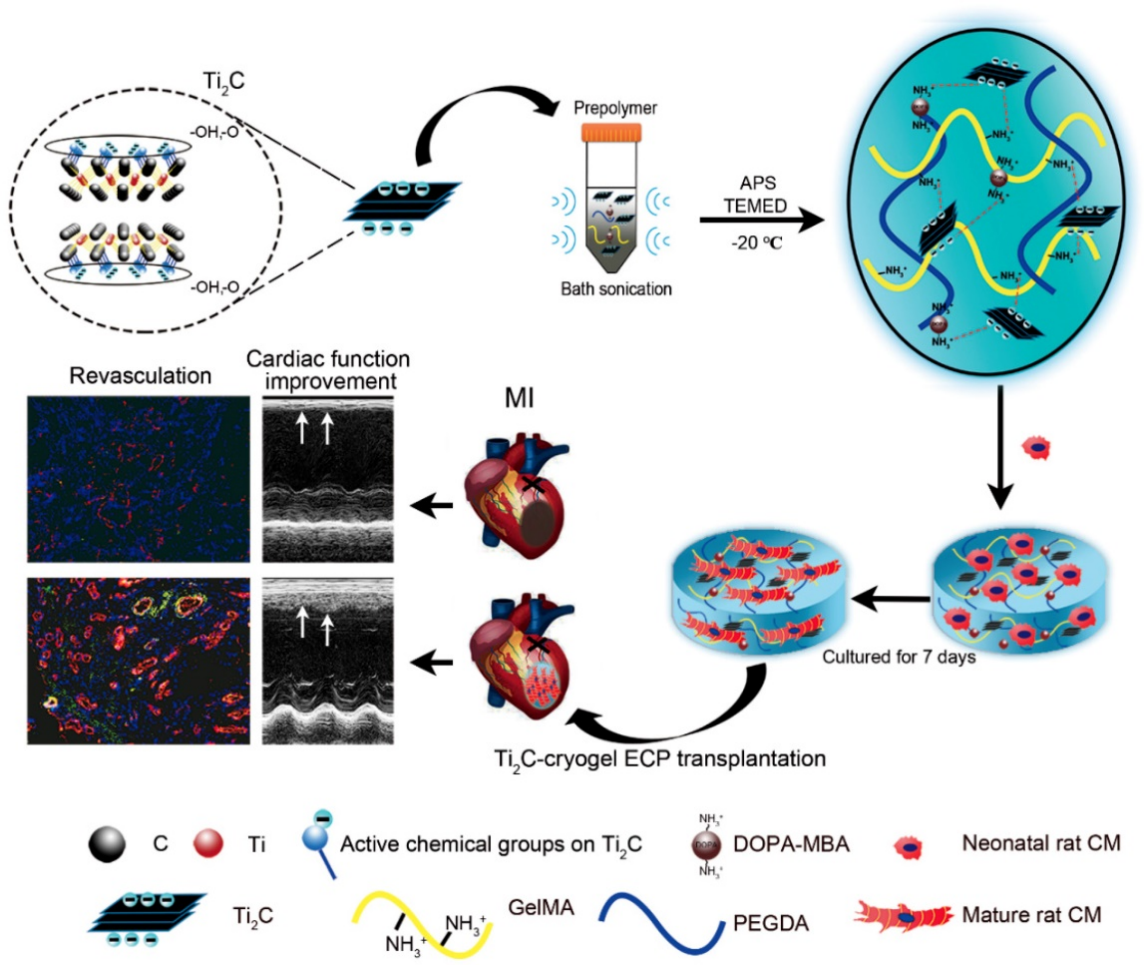
** Schematic illustration of the fabrication of Ti_2_C-cryogel and its application in a rat MI model.** After etching with HF the MAX phase Ti_2_AlC was transformed into the MXene phase Ti_2_C. The Ti_2_C nanoparticle was added to the prepolymer solution via bath sonication, then the Ti_2_C-cryogel was fabricated through chemical crosslinking at -20 °C. Finally, the Ti_2_C-cryogel ECPs were transplanted onto the infarct area to repair MI.

**Figure 1:**
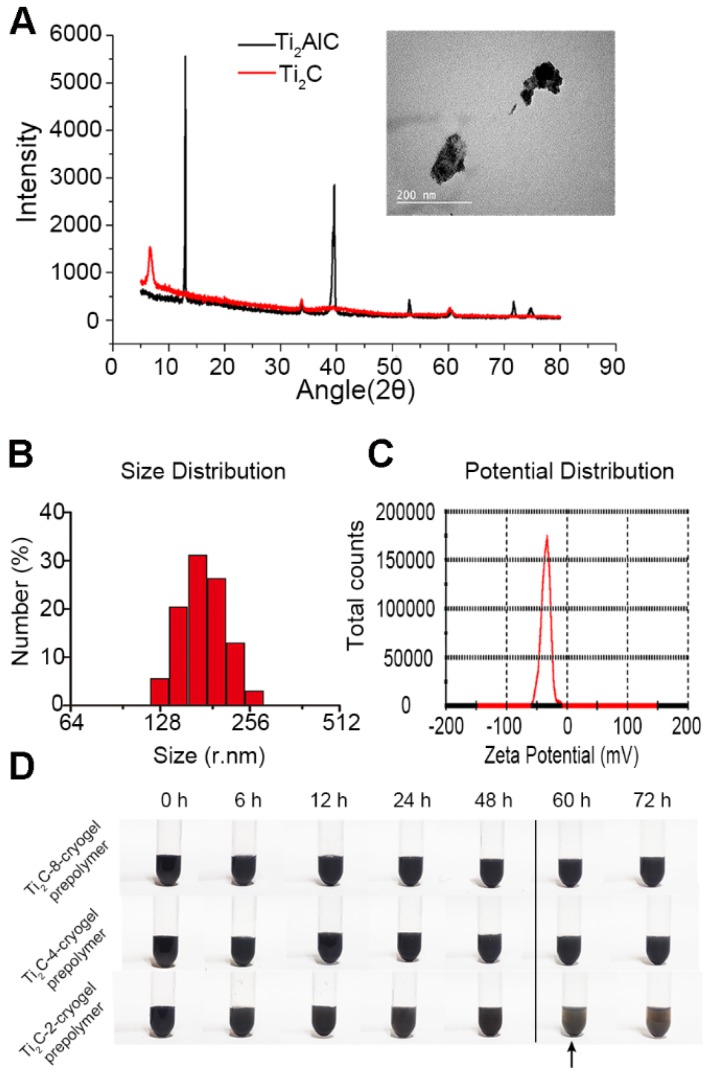
** Characterization of the Ti_2_C nanoparticles.** A) XRD analysis for MAX Ti_2_AlC and MXene Ti_2_C. The inset shows the representative TEM image of Ti_2_C nanoparticles. B) Size distribution and C) Zeta potential distribution of Ti_2_C nanoparticles dispersed in deionized water. D) Different Ti_2_C-cryogel prepolymer solutions were placed for 72 hours. Arrow showed a slight aggregation in the Ti_2_C-2-cryogel prepolymer solution.

Herein, we first introduce MXene Ti_2_C into cryogel to construct a functional ECP for MI repair (Scheme 1). Firstly, Ti_2_C was synthesized by treating the MAX phase Ti_2_AlC with hydrofluoric acid (HF) 46. Powder X-ray diffraction (XRD) clearly showed that diffraction peak at 13° 2θ in Ti_2_AlC was broadened and shifted to 8° 2θ after HF treatment and revealed the expansion of the interlayer and the successful removal of the Al layers (Figure 1A) 21. Under the TEM fields, the synthesized Ti_2_C displayed the flake structures (Figure 1A), just as the previous report 28. In addition, the DLS analysis showed that the prepared Ti_2_C particles were negatively charged and reached the nanoscale with an average size of 181.52 nm (Figure 1B and C). Owing to the hydrophilic groups and the electrostatic adsorption, Ti_2_C nanoparticle could keep uniform dispersion in the prepolymer during the cryogelation process. Only a slight aggregation of nanoparticles in the prepolymer solution occurred when the prepolymer stayed at room temperature for more than 60 hours (Figure 1D). While for the traditional conductive material such as graphene oxide, the aggregation happened after the prepolymer stayed at room temperature for 12 hours (Figure S1).

Although the low cytotoxic effect of Ti_2_C MXene on human skin-derived cell lines HaCaT has been confirmed 28, its cytotoxic effect on the cardiomyocytes is still unclear. In this regard, we first analyzed the biocompatibility of Ti_2_C nanoparticles to CMs. After being treated with 25 μg/ml, 100 μg/ml and 250 μg/ml Ti_2_C nanoparticles for 1 day and 3 days respectively, cells live-dead staining showed that few red cells (dead cells) were detected among all the groups (Figure S2A). The quantitative analysis of the green cells (living cells) showed that there was no difference in the proportion of living cells between the treated cells and untreated cells, suggesting the minimized cytotoxicity of Ti_2_C nanoparticles for CMs (Figure S2B). Some studies reported that Ti_2_C nanoparticles could inhibit tumor cell growth through inducing ROS production in some tumor cells 28. Whether Ti_2_C nanoparticles induced the ROS production in CMs could be a potential unfavorable risk. While after the CMs treated with Ti_2_C nanoparticles for 3 days, the ROS detection showed that low fluorescence was detected among with or without nanoparticles treated groups, only the high fluorescence was detected in the H_2_O_2_ treated group (Figure S3B). Under the TEM filed, only a few Ti_2_C nanoparticles were englobed into the CMs while the cellular morphology and cell state was no difference compared with the CMs without Ti_2_C nanoparticles treatment (Figure S3A). Taken together, the Ti_2_C nanoparticles would not damage the CMs.

**Figure 2:**
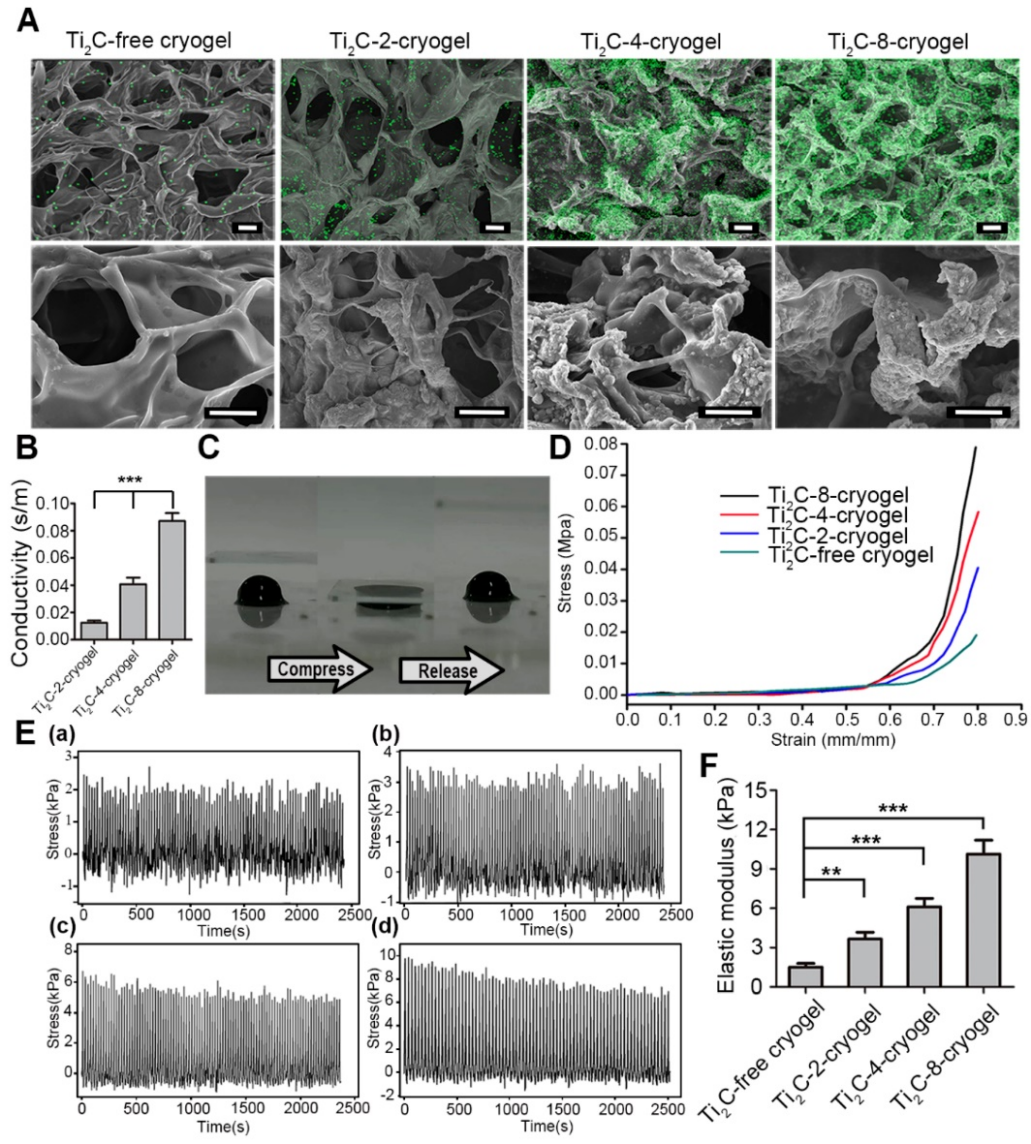
** Characterization of the Ti_2_C-free cryogel and the different Ti_2_C-cryogels.** A) Atomic distribution maps (up) and SEM images (bottom) of all the cryogels showed that the Ti_2_C nanoparticles well distributed on the inside wall of the porous cryogels in different Ti_2_C-cryogel groups. Green spots represented the Ti element. Scale bars (up)= 50 μm, Scale bars (bottom) = 25 μm. B) Electrical conductivities of the different Ti_2_C-cryogels. n = 5. ***p<0.001. C) Excellent-resilience of the Ti_2_C-8-cryogel. D) Stress-strain curves of the different cryogels. n = 3. E) The stress-time curve of different cryogels compressive cycling test up to 60 % deformation for 100 cycles. F) Elastic modulus of the different cryogels. n = 3. **p<0.01, ***p<0.001

Therefore, to test the feasibility of Ti_2_C nanoparticles in myocardial tissue repair, we proposed to introduced the conductive Ti_2_C into scaffold materials to construct a conductive ECP for MI repair. Additionally, our previous developed mussel-inspired cryogel has been proved to be beneficial for CMs adhesion and maturation and could be taken as the ideal support for MI repair. Here, the Ti_2_C could be well distributed into the cryogel prepolymer through electrostatic adsorption between negatively charged Ti_2_C nanoparticles and positively charged cryogel polymer (free amino groups in dopamine 50 and residual amino groups in Gel-MA 41) via simple water bath sonication. As shown in Figure 2A, Ti_2_C nanoparticles tightly attached to the inner wall in the cryogel, endowing the Ti_2_C-cryogel with a coarse surface which is beneficial for cell attachment and spreading 34. The energy-dispersive X-ray spectroscopy (EDX) in the randomly selected area further confirmed the presence of Ti_2_C within the cryogel resulted in a rough surface, and the atomic ratio of titanium element even reached to 36% in Ti_2_C-8-cryogel group (Figure S4).

Previous studies proved that the macroporous structure in cryogel was beneficial for cell communication and nutrient delivery, thus promoting cell proliferation and spreading 51, 52. As shown in Figure 2A and S4 B**,** the average pore size was 53.84 ± 5.53 μm in the Ti_2_C-free cryogel, 53.52 ± 7.08 μm in the Ti_2_C-2-cryogel, 52.37 ± 7.54 μm in the Ti_2_C-4-cryogel and 58.37 ± 10.29 μm in the Ti_2_C-8-cryogel respectively. The introduction of a proper portion of Ti_2_C nanoparticles would not alter the pore size of the cryogels.

The mechanical property of the hydrogel scaffold has played a vital role in ECP construction. An ideal scaffold for ECP should possess two mechanical properties, firstly, an appropriate elastic modulus that is close to the natural heart can serve for the synchronous contraction of cardiomyocytes *in vitro* 53, 54, and secondly, an excellent fatigue resistance can support steady cardiac systole and diastole after it is transplanted onto the infarct heart *in vivo*. In this regard, we conducted a cycling compressive test on different cryogels. All cryogels did not get rupture after compressing to 60% strain. Moreover, introducing the Ti_2_C nanoparticles could significantly enhance the elastic modulus and mechanical strength of the cryogel. The mechanical strength increased from 2.24 kPa to 9.65 kPa in a 60% compress strain after compounding 8 mg/ml Ti_2_C. And the elastic modulus of Ti_2_C-8-cryogel was 10.13 ± 1.06 kPa which was close to that of nature myocardium during heart diastole 44. Furthermore, the stress-time curves and the stress-strain curves of the Ti_2_C-4-cryogel and Ti_2_C-8-cryogel exhibited more steady than the Ti_2_C-free cryogel and Ti_2_C-2-cryogel (Figure 2D-F and Figure S3 D).

Combined with the SEM results, the Ti_2_C nanoparticles would not change the macroporous structure of Ti_2_C-cryogel which is vital for the entire cryogel to release stress when got compression. As shown in Figure 2C and movie S1, the Ti_2_C-8-cryogel could be compressed easily and recover the original shape quickly. Furthermore, the uniform contribution of Ti_2_C nanoparticles strengthened the wall of the pores in Ti_2_C-cryogel so that the Ti_2_C-cryogel exhibited better toughness compared to Ti_2_C-free cryogel under cycle compression. Thus the Ti_2_C nanoparticles could enhance not only the mechanical strength but also the toughness and the fatigue resistance of the cryogel.

The conductivity of Ti_2_C-cryogel was detected using a four-point probe measurement. Ti_2_C endowed the cryogel with semi-conductive properties. The conductivity of the Ti_2_C-cryogel was depended on the concentration of the Ti_2_C (Figure 2B). Abnormal electric conduction in the heart after MI and anomalously-conductive grafts transplantation may result in severe cardiac arrhythmia incidents 44, 11, so it is a key issue to mimic the conductivity of native heart in ECP construction. In our result, the conductivity of the Ti_2_C-8-cryogel reached 0.087 S/m that was equivalent to that of the natural heart 43.

In consideration of crosslinking MA with Ti_2_C could endow Ti_2_C with more chemical functional groups, we have fabricated MA-Ti_2_C using the crosslinking method. The FTIR Spectrum of MA-Ti_2_C (Figure S4A) showed that the peak near 1081 cm^-1^, 1527 cm^-1^, and 1750 cm^-1^corresponded to -C-O-, C=C, and C=O 55. The peak near 563 cm^-1^ and 1634 cm^-1^, which corresponded to the Ti-O-Ti and Ti-C bone 56, could be detected in both of MA-Ti_2_C and Ti_2_C spectrum. The FTIR spectrum confirmed that the methacrylic acid had been cross-linked with Ti_2_C via esterification between methacrylic acid and -OH of Ti_2_C. However, the MA-Ti_2_C derived cryogel exhibited very low conductivity compared to the prepared high conductive Ti_2_C-cryogel mentioned above (Figure S4B). This probably owing to the esterification that could affect functional groups such as -OH in Mxene Ti_2_C. Some theoretical studies explained that functional groups play an important role in increasing the electrical properties of Mxenes 57. Overall this Ti_2_C-cryogel fabricated via simple sonication was appropriate to be used for the conductive ECP construction.

### Cell viability and maturation of CMs seeded on Ti_2_C-cryogel ECP* in vitro*

**Figure 3:**
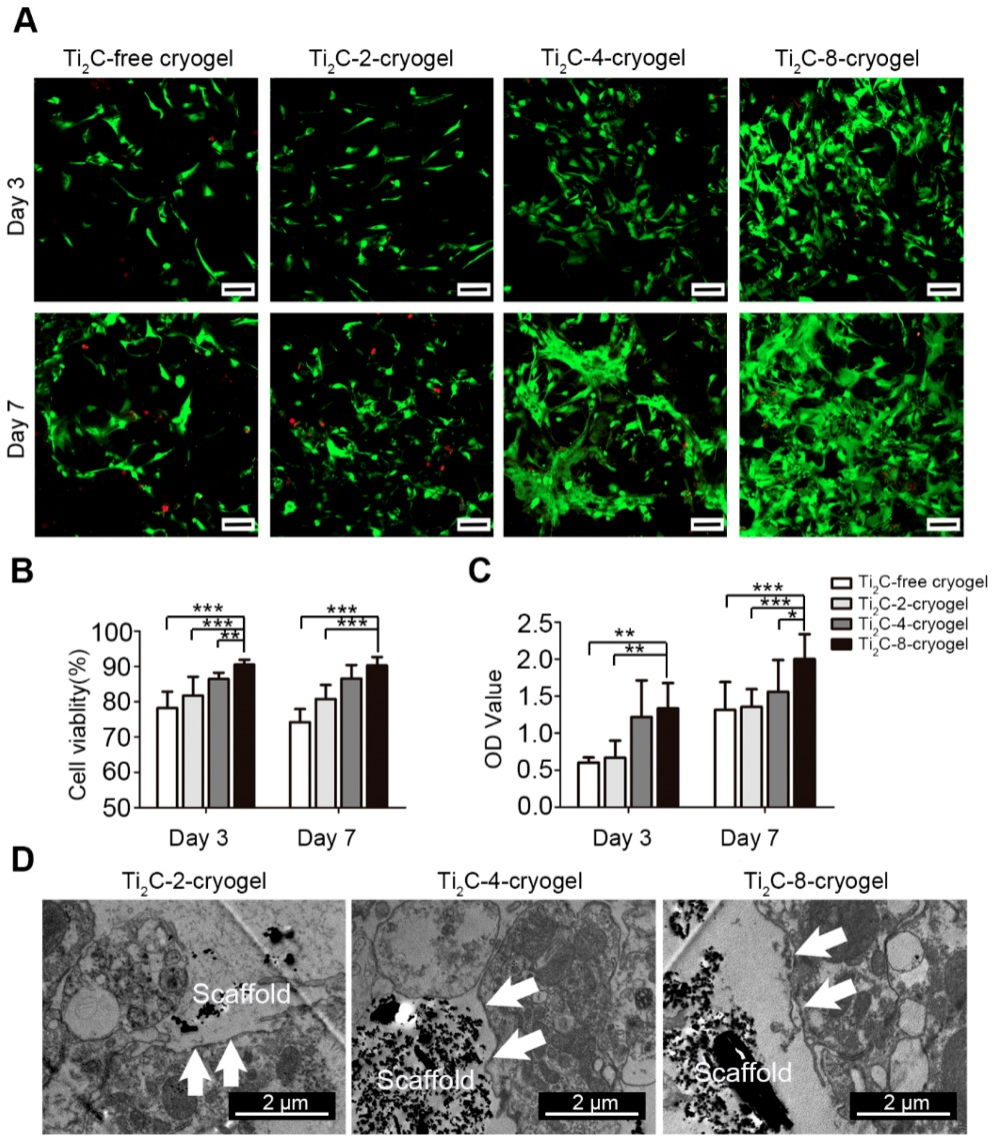
** Biocompatibility of the Ti_2_C-cryogels.** A) Live-dead staining for CMs on different Ti_2_C-cryogels on the day 3 and day 7 of culture Scale bars = 100 μm.B) Quantitative cell viability of CMs based on the live-dead staining. n = 3. **p<0.01, ***p<0.001 C) CCK-8 assay for CM cultured on different Ti_2_C-cryogel on day 3 or day 7. n = 6. *p<0.05, **p<0.01, ***p<0.001 D) Low magnification TEM images of CMs cultured on different Ti_2_C-cryogels showed that the Ti_2_C nanoparticles distributed on the surface of CMs, not inside them. Arrows showed the membranes of CMs.

**Figure 4:**
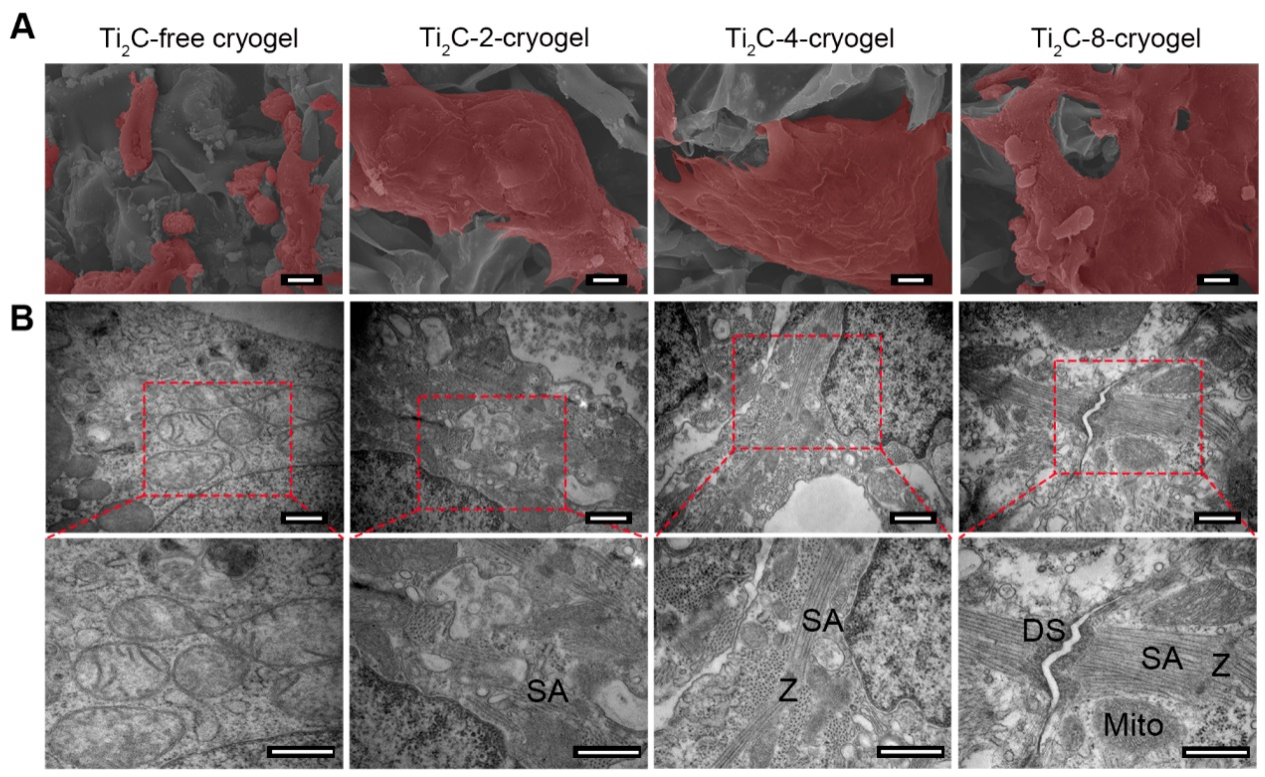
** Ultrastructure of the CMs cultured on the Ti_2_C-free cryogel ECP and different Ti_2_C-cryogel ECPs on day 7 of culture.** A) Pseudo-color SEM images showed the morphology of CMs in the different ECPs. Scale bars = 10 μm B) TEM images showed the ultrastructure of CMs in the different ECPs. Sarcomeres (SA), Z-lines (Z), mitochondria (Mito) and desmosomes (DS) were observed in CMs on the Ti_2_C-cryogel ECPs. Scale bars = 500 nm.

Cell viability of the CMs cultured on Ti_2_C-free cryogel and Ti_2_C-cryogel (Ti_2_C-2-cryogel, Ti_2_C-4-cryogel, and Ti_2_C-8-cryogel) were determined by cell live-dead staining and CCK-8 assay at day 3 and day 7. Compared with those in the Ti_2_C-free cryogel, the number of living cells (green) in Ti_2_C-4-cryogel and in Ti_2_C-8-cryogel had been calculated more (Figure 3A). On day 7 of culture, the percentage of living cells in Ti_2_C-8-cryogel was more than 90%, while that in Ti_2_C-free cryogel was only 74% (Figure 3B). The excellent cell viability of CMs in Ti_2_C-8-cryogel was also confirmed by CCK-8 assay (Figure 3C). In addition, after culturing for 7 days, well spreading and continuous CMs on Ti_2_C-cryogel (especially on the Ti_2_C-8-cryogel) were observed under SEM while CMs maintain the globular shapes on the Ti_2_C-free cryogel (Figure 4A). This excellent biocompatibility of the Ti_2_C-cryogel might attribute to the following reasons. Firstly, dopamine-MBA in the Ti_2_C-cryogel could act as an affiant to help cells adhesion and retention via the dopamine's adhesive capability 40. Secondly, dopamine-MBA also could absorb surrounding proteins, which was beneficial for cell attachment 58, 59. Thirdly, the coarse surface in the Ti_2_C-cryogel (especially in Ti_2_C-8-cryogel) could promote cell attachment and spread 34, 60. In addition, ultrastructure observation indicated that most of the negative Ti_2_C nanoparticles merely distributed on the negative cell surface of CMs, endowing them with excellent biocompatibility (Figure 3D).

In order to assess the maturation of CMs on Ti_2_C-cryogel, the ultrastructure of CMs cultured on day 7 was observed under TEM (Figure 4B). Packed strong sarcomeres and clear Z-line structures, which typically represented robust CMs structures, were observed in CMs cultured both on Ti_2_C-4-cryogel and Ti_2_C-8-cryogel. A few of weak and disorganized sarcomeres formed in CMs cultured on Ti_2_C-2-cryogel, while few sarcomere structures could be detected in the Ti_2_C-free cryogel group. Large segments of desmosomes in corresponding to that in native myocardium were observed in Ti_2_C-8-cryogel group under TEM 61. This implied that the CMs cultured on Ti_2_C-8-cryogel could form initial intercalated discs. The formation of distinct intercalated discs is an important sign of myocardial maturation 62.

**Figure 5:**
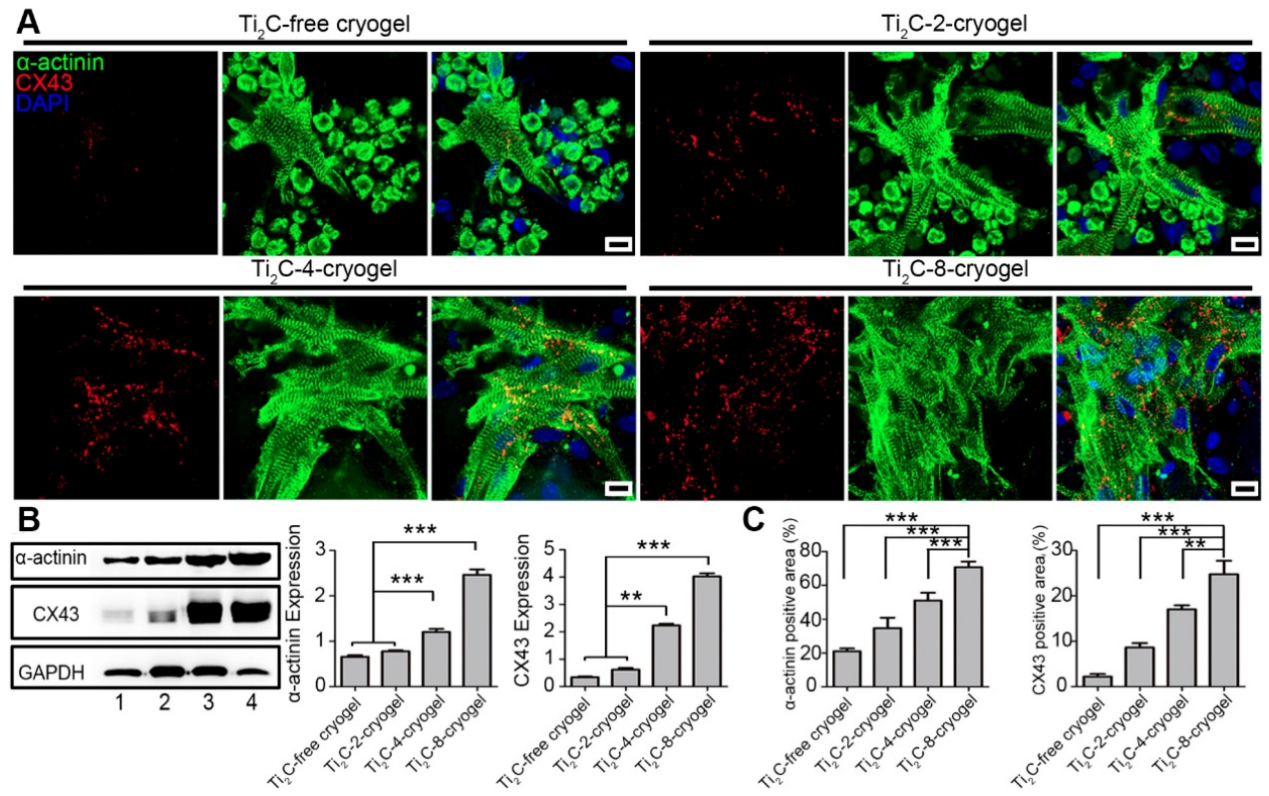
** Expression of cardiac-specific proteins in CMs cultured on different ECPs on day 7**. A) Immunofluorescence images of α-actinin and CX-43 proteins of CMs on different cryogels on day 7. Scale bars = 10 μm. B) Western blotting and the quantification for the expressions of α-actinin proteins and CX-43 proteins in CMs on different ECPs on day 7 of culture. Line 1: Ti_2_C-free cryogel. Line 2: Ti_2_C-2-cryogel. Line 3: Ti_2_C-4-cryogel. Line 4: Ti_2_C-8-cryogel. n = 3. **p < 0.01, ***p < 0.001. C) α-actinin and CX43 positive area coverages of CMs based on the immunofluorescence images. n = 4. **p < 0.01, ***p < 0.001.

Both sarcomeric α-actinin and CX43 proteins are molecular markers of the mature CMs. Hereinto, α-actinin is prime microfilament protein for myocardium contraction 63, while CX43 is a well-known gap junction protein between myocardiocytes 64. On day 3 of culture, the CMs on Ti_2_C-free cryogel presented round shape with few intact sarcomeres, while the CMs on Ti_2_C-4-cryogel and Ti_2_C-8-cryogel exhibited an extended structure with stripe-shaped sarcomeres under immunostaining (Figure S7). On day 7 of culture, a higher expression of α-actinin, and more evident sarcomeres were found in CMs on Ti_2_C-8-cryogel compared to those on Ti_2_C-2-cryogel or Ti_2_C-free cryogel. Dense CX43 proteins along with the cardiomyocytes membrane between CMs obviously presented in Ti_2_C-8-cryogel (Figure 5A). Coverage of the α-actinin positive area and the CX43 positive area were significantly improved in the Ti_2_C-cryogel groups compared to those in the Ti_2_C-free cryogel group (Figure 5C). Similar results also confirmed that the protein level of α-actinin in the Ti_2_C-8-cryogel group was more than twice and the CX43 level in the Ti_2_C-8-cryogel group was proximity four times than that in Ti_2_C-free cryogel group using western blot assays (Figure 5B).

Overall, our studies suggested that the cryogel combined with the appropriated concentration of Ti_2_C (4 mg/ml or 8 mg/ml) significantly improved cell attachment and spreading. The Ti_2_C-8-cryogel scaffold exhibited excellent abilities to facilitate intercellular connection and to accelerate CMs maturation.

### Intracellular Ca^2+^ transient and beating behavior of CMs on Ti_2_C-cryogel ECP

**Figure 6:**
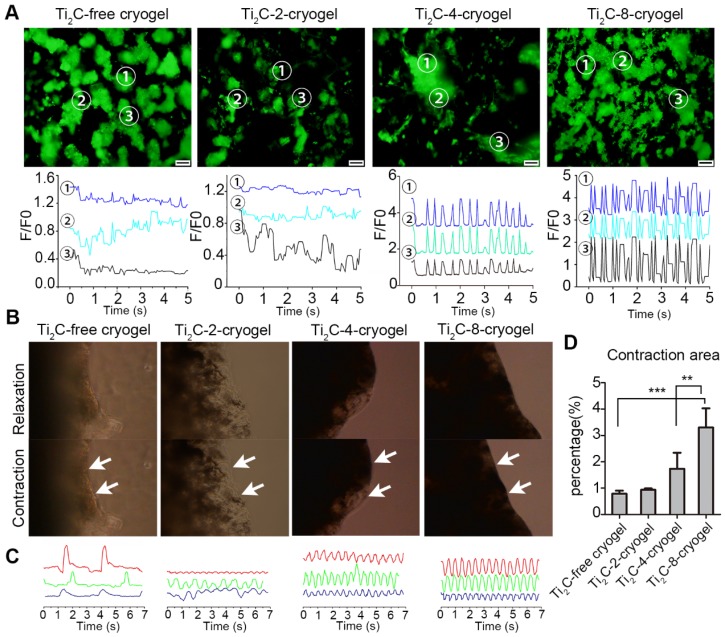
** Calcium transient analysis and the contraction behavior for the CMs on Ti_2_C-free cryogel ECP and different Ti_2_C-cryogel ECPs.** A) Calcium transient (up) and extracted related frequency signals (bottom) in CMs cultured on different ECPs for 3 days. B) Photographs of different ECPs cultured for 7 days displayed contraction behavior under a microscope field. C) Beating signal graphs represented the most stable contraction in Ti_2_C-8-cryogel ECP among all the ECPs. Each line of the beating signal represented one sample of each group. D) Quantified contraction area of all the ECPs based on the photographs from a microscope. n = 3. **p < 0.01, ***p < 0.001. Scale bars = 200 μm.

To investigate whether the Ti_2_C-free cryogel or the Ti_2_C-cryogel could promote the electrical conduction between cardiomyocytes, the intracellular Ca^2+^ transient propagation of CMs on different cryogels were measured via Ca^2+^ indicator Fluo-4 Am, and the fluorescence signal was recorded with real-time video on day 3. At three different randomly selected spots, strong, high-frequency and synchronous Ca^2+^ puffs were observed in the Ti_2_C-8-cryogel group. On the contrary, weak and asynchronous Ca^2+^ puffs occurred in the Ti_2_C-free cryogel group (Figure 6A and Movie. S2). These results indicated that the strong Ca^2+^ puffs of CMs were related to the dose of Ti_2_C in cryogel ECP. Strong and rhythmic Ca^2+^ puffs facilitated the synchronous contraction of CMs on Ti_2_C-8-cryogel. The mature CMs seeded on this excellently conductive and elasticity-suitable cryogel could be taken as a functional ECP with the spontaneous rhythmic beating. High concentration Ti_2_C (such as that in Ti_2_C-4-cryogel and Ti_2_C-8-cryogel) in cryogel could facilitate electrically signal transmission between CMs. On day 3 of culture, spontaneous contraction of ECPs could be observed in all groups under the microscopic field, while larger contraction amplitude could be observed in the Ti_2_C-4-cryogel ECP and the Ti_2_C-8-cryogel ECP compared to that in the Ti_2_C-2-cryogel ECP and the Ti_2_C-free cryogel ECP (Figure 6B and C, Movie. S3). The contraction amplitude of the Ti_2_C-8-cryogel ECP reached approximately three times more than that of the Ti_2_C-free cryogel ECP. The beating signal pattern recorded with real-time video under the microscope indicated that Ti_2_C-8-cryogel ECP possessed rhythmic, high-frequency and stable beating, while Ti_2_C-2-cryogel and Ti_2_C-4-cryogel ECP exhibited irregular beating. Among them, the Ti_2_C-free cryogel ECP merely exhibited quite low-frequency beating. On day 7 of culture, the spontaneous beating of the Ti_2_C-8-cryogel ECP became very strong and could be observed even in naked eyes (Movie. S4). This high-frequency and holistic beating behavior mainly contributed to the formation of tightly connected mature CMs multilayers in our developed functional Ti_2_C-cryogel ECP. We supposed that our developed conductive ECPs could bridge the healthy myocardium areas across the scar region and help maintain normal electrical propagation after MI 19.

### Ti_2_C-cryogels induce tube structure formation of the endothelial cells

To assess the angiogenesis potential in the Ti_2_C-cryogels. RAECs were cultured in Ti_2_C cryogel for 3 days and stained with F-actin phalloidin to observe the RAECs vasculation. As Figure S8 showed, a vessel-like structure formed in Ti_2_C-cryogels, and interestingly, some 3D tube structure could be found in the Ti_2_C-8-cryogel. Furthermore, the expression of the vasculation related gene eNOS and VEGF was evaluated by the qPCR analysis. Both eNOS and VEGF gene expression in the Ti_2_C-8-cryogel group were significantly higher compared with the 2D glass slide group and Ti_2_C-free cryogel group (Figure S8 B and C) There are some conductive materials such as gold rod, carbon nanotube, and Ti_2_O reported that could promote the vessel formation. These studies reported that conductive materials could enhance the cell communication between endothelial cells, promote the migration of endothelial cells to form a tube-like structure 65-67. In our study, the high expression of the angiogenetic mRNA in the Ti_2_C-cryogel groups indicated that the conductive Ti_2_C nanoparticle could play an important role in the vasculation of RAECs. In addition, compared with the 2D culture condition, the 3D microenvironment of the Ti_2_C-cryogel could also provide a more physiological condition for endothelial cells to form vessels-shape frameworks.

### Repairs of Ti_2_C-cryogel ECP for MI in the rat model

**Figure 7:**
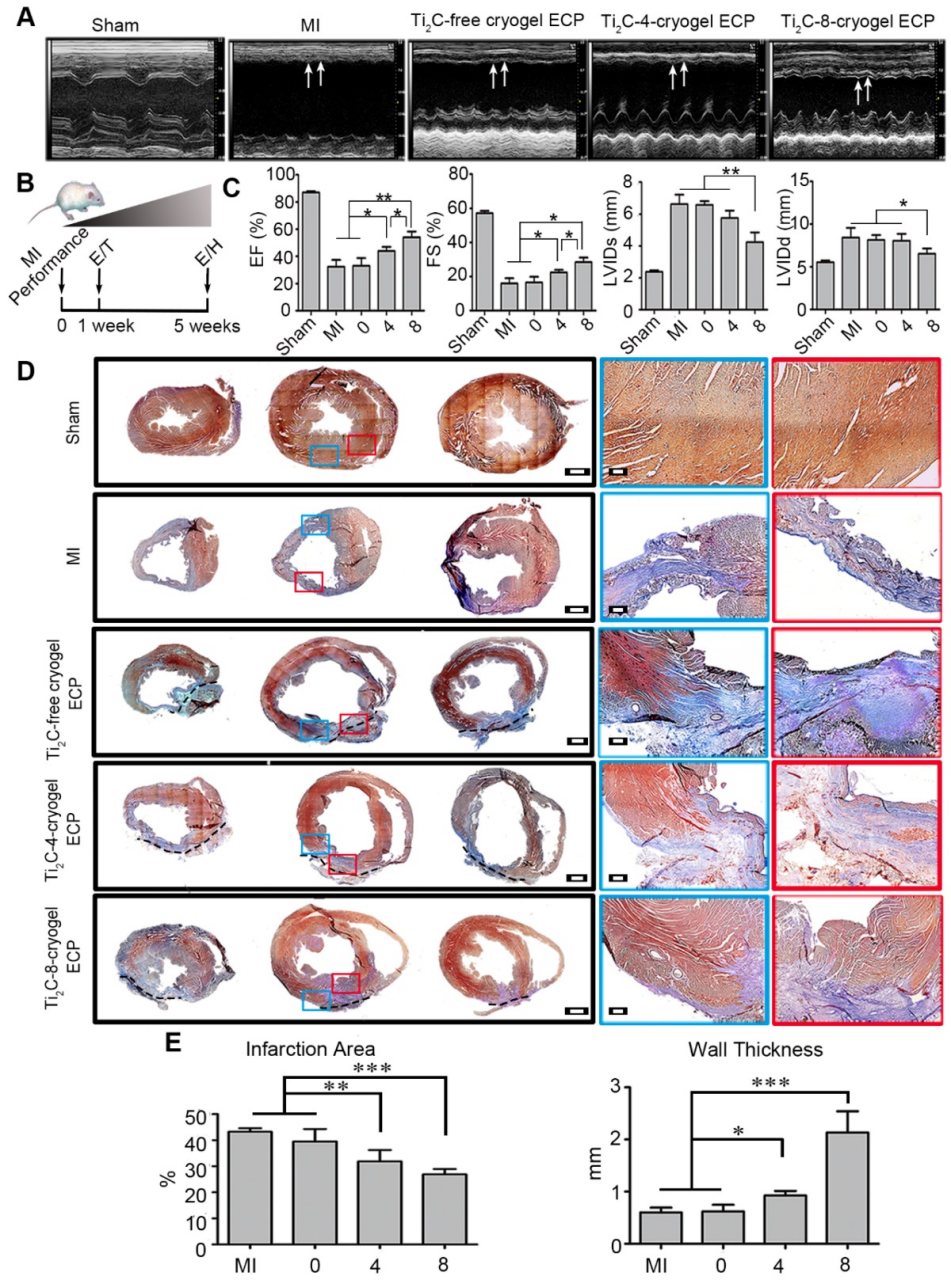
** Echocardiography evaluation and pathological analysis after Ti_2_C-cryogel ECPs transplantation for 4 weeks.** A) Representative echocardiographic images of different groups. Arrows showed the contractions of LV anterior walls. B) Experimental design. 1 week after MI performance, rats with FS lower than 30% were selected and performed ECP transplantation (T). The effects of the ECP were analyzed by echocardiography (E) and histology (H) at 4 weeks after transplantation. C) Typical echocardiographic parameters of the left ventricular function for different groups. n=3 *p < 0.05, **p < 0.01. D) Masson's trichrome staining for multiple heart sections from apex to the atrium in different groups. Blue staining represented fibrous tissue and red staining represented myocardium. Scale bars = 1 mm at low magnification, Scale bars = 200 μm at high magnification. E) Quantitative analysis of wall thickness and infarct area of left ventricular anterior wall based on the images of Masson's trichrome staining. n = 3. *p < 0.05, **p < 0.01, ***p < 0.001. 0 represented the Ti_2_C-free cryogel ECP group, 4 represented the Ti_2_C-4-cryogel ECP group, and 8 represented the Ti_2_C-8-cryogel ECP group.

Considering the exceptional effect of Ti_2_C-cryogel ECPs in facilitating cardiomyocytes maturation and RAEC tube structure formation* in vitro*, we further assessed the therapeutic efficacy of the developed Ti_2_C-cryogel ECPs for MI *in vivo*. After being cultured for 7 days *in vitro*, the different ECPs were transplanted onto the infarct region in MI rats for 4 weeks. The echocardiographic images and the short-axis B model echo video (Movie. S5) showed that bare contraction occurred in the left ventricular anterior wall in the MI group or the Ti_2_C-free cryogel ECP transplantation group, while the obvious contractile activity of the left ventricle anterior wall was observed in the Ti_2_C-8-cryogel ECP transplantation group (Figure 7A). Typical echocardiography parameters, EF, FS, LVIDs, and LVIDd which represented the contraction function of the left ventricle were also analyzed 49. Both EF and FS were significantly elevated in the Ti_2_C-8-cryogel ECP transplantation group and the Ti_2_C-4-cryogel ECP transplantation group compared to those in the MI group. Among them, the Ti_2_C-8-cryogel ECP transplantation group possessed the highest EF and FS values (Figure 7C). These results suggested that Ti_2_C-8-cryogel ECP could improve the cardiac function in MI rats. Based on the echocardiography parameters, there were no differences between the MI group and the Ti_2_C-free cryogel ECP transplantation group.

After transplantation for 4 weeks, the transplanted ECPs were observed to well adhere to the surface of epicardium. Masson's trichrome staining showed that most of the left ventricle anterior wall was occupied with the blue staining fibrous tissues, and bare red staining myocardium tissues were detected in the infraction region in the MI group, whereas more regenerative myocardium tissues stained with red were observed in the Ti_2_C-4-cryogel group and the Ti_2_C-8-cryogel group (Figure 7D). The infarct area percentage analysis through Masson's trichrome staining images showed that the least infarct area was appeared in the Ti_2_C-8-cryogel ECP group (26.88%) among all groups. The infarct area percentages of the Ti_2_C-4-cryogel ECP group, Ti2C-4-cryogel ECP group and MI group were 31.84%, 39.51%, and 43.22%, respectively. Additionally, the left ventricular wall thickness of the Ti_2_C-8-cryogel ECP group reached 2.13 mm, while that of the MI group was only 0.6 mm. Masson's trichrome staining for the multiple sections of the same heart showed that most fibrotic tissue of heart was in the apex and few scars were found in upper sections in the Ti_2_C-8-cryogel ECP group while the fibrotic tissue occupied most of the whole ventricle in MI group. These results suggest that the Ti_2_C-8-cryogel ECP could enhance the cardiac function and reduce scar formation in MI rats.

### Ti_2_C-cryogel ECP could reduce inflammation and establish a favorable microenvironment in the infarcted region for myocardial regeneration

**Figure 8:**
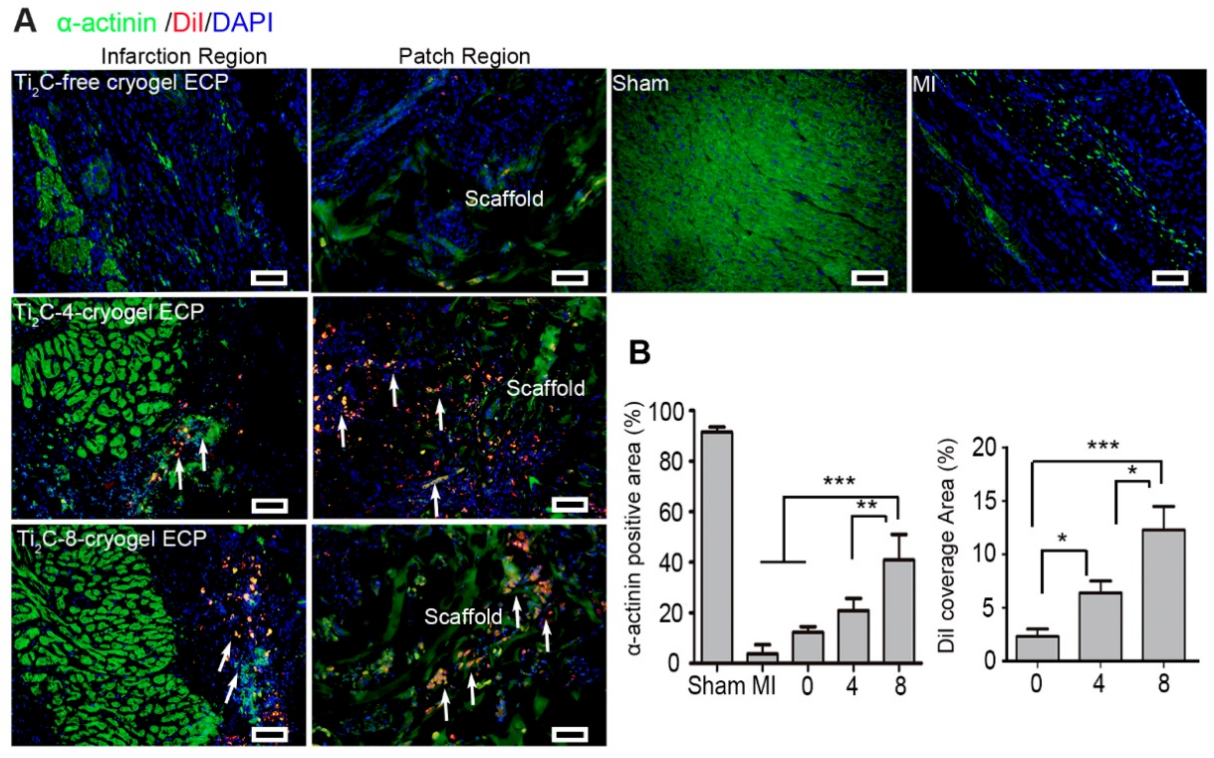
** Ti_2_C-cryogel ECP promoted the repair of damaged myocardium.** A) Cardiac-specific marker α-actinin expression in the infarct region and patch region of the heart in different groups via immunostaining. Arrows showed the DiI-stained CMs. scale bars = 50 μm B) Corresponding α-actinin positive area and the DiI-labeled CMs coverage area based on the immunostaining images. n = 3. *p < 0.05, **p < 0.01, ***p < 0.001. 0 represented the Ti_2_C-free cryogel ECP group, 4 represented the Ti_2_C-4-cryogel ECP group, and 8 represented the Ti_2_C-8-cryogel ECP group.

To further evaluate the role of the Ti_2_C-cryogel ECP for myocardial regeneration, immunofluorescent staining was performed on myocardial sections using cardiac marker α-actinin after ECPs transplantation for 4 weeks. Figure 8A showed in the Ti_2_C-8-cryogel ECP transplantation group, green α-actinin occupied most of the infarction region. In addition, the diI-labeled allogeneic CMs were not only resided in the ECP scaffold but in the infarction region with the expression of also the myocardial mature marker α-actinin. (Figure 8A). The quantitative index revealed that α-actinin positive area in the Ti_2_C-8-cryogel ECP transplantation group was approximate 2-fold higher than that in the Ti_2_C-4-cryogel ECPs transplantation group and 4-fold higher than that in the MI group. And the DiI coverage area from the scaffold region to the infarction region also indicated that the survived allogeneic CMs were most in the Ti_2_C-8-cryogel ECP transplantation group (Figure 8B).

**Figure 9:**
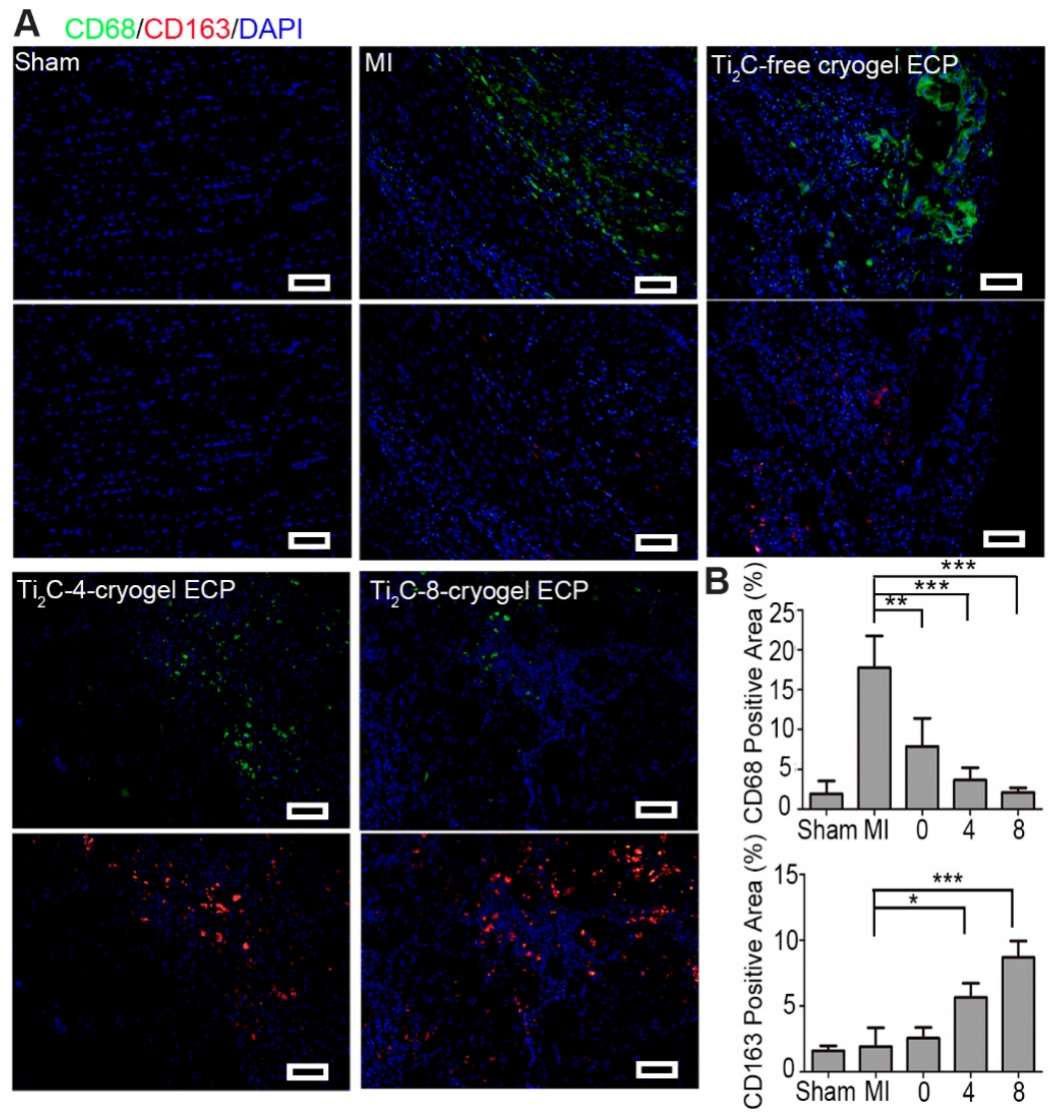
** Ti_2_C-cryogel ECP induced pro-healing M2 macrophage polarization in the MI region.** A) Expression of M1 macrophage-specific marker CD68 (green) and M2 macrophage-specific marker CD163 (red) in the MI region after 28 days of transplantation. scale bars = 50 μm. B) Quantification of the corresponding CD68 and CD163 level. n = 3. *p < 0.05, **p < 0.01, ***p < 0.001. 0 represented the Ti_2_C-free cryogel ECP group, 4 represented the Ti_2_C-4-cryogel ECP group, and 8 represented the Ti_2_C-8-cryogel ECP group.

Previous studies have demonstrated that favorable cardiac microenvironment was a key issue in cardiac survival and regeneration 68, 69, while inflammatory reaction as a major component of early MI microenvironment was thought to be detrimental to cardiac repair 70. Thereinto, macrophages were recruited and released inflammatory factors such as transforming growth factor (TGF-β), which activated profibrotic processes and further promoted the scar formation. Herein, we investigated whether the repair effect of the developed Ti_2_C-cryogel ECP is related to the inflammatory microenvironment. In the infarction region of the MI rats, F4/80 and CD68 positive pro-inflammatory macrophages would assemble and induce an unfavorable inflammatory response and accelerate the fibrosis. Our results showed that expression of F4/80 and CD68 were lower in Ti_2_C-cryogels groups than the MI group indicated that few M1 macrophages scattered in the infarct region in the Ti_2_C-8-cryogel ECP transplantation group (Figure 9A and S9). Although the reduced levels of F4/80 proteins were also observed in the Ti_2_C-free cryogel ECPs group and the Ti_2_C-4-cryogel ECPs group, the Ti_2_C-8-cryogel ECPs group presented the lowest level of F4/80 proteins as shown in Figure S9. Comparatively, the expression of pro-healing M2 macrophages specific marker CD163 was significantly higher in the Ti_2_C-8-cryogel ECP transplantation group than the MI group (Figure 9B). Obviously, in our developed Ti_2_C-8-cryogel ECPs-based microenvironment, these evidently decrease of proinflammatory and pro-fibrogenic macrophages and the recruitment of pro-healing macrophages could be greatly beneficial for CMs surviving and MI repair.

### Ti_2_C-cryogel ECP promoted myocardial repair by enhancing angiogenesis

**Figure 10:**
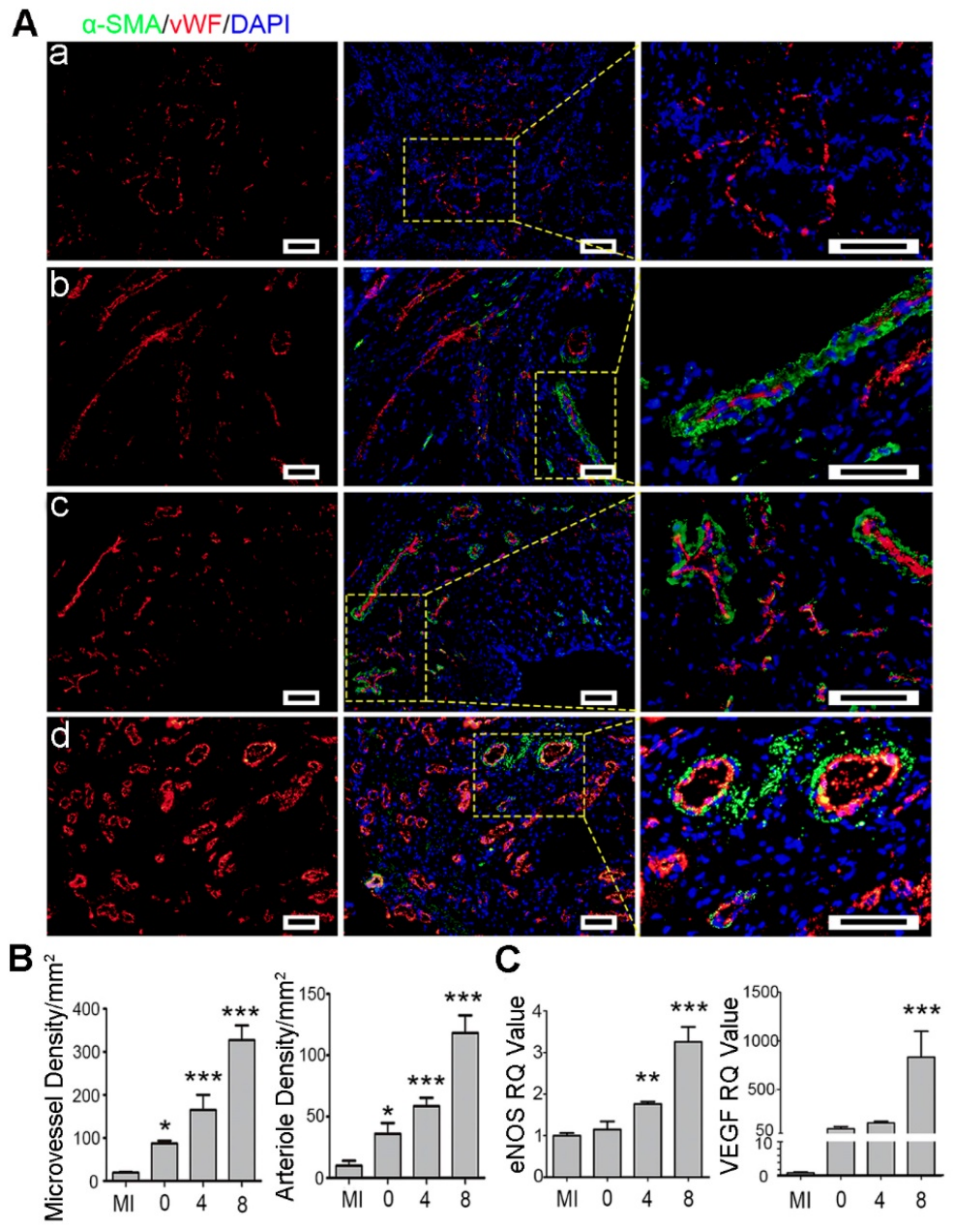
** Ti_2_C-cryogel ECP promoted revasculation in the infarcted heart.** A) Typical vascular endothelial cell proteins (vWF) and vascular smooth muscle cell proteins (α-SMA) expression in infarct region of the heart in different groups, including MI group (a), Ti_2_C-free cryogel ECP group (b), Ti_2_C-4-cryogel ECP group (c), and Ti_2_C-8-cryogel ECP group (d). Scale bars = 50 μm. B) Statistical analysis of microvessels and arterioles densities in different groups. C) Expression of eNOS and VEGF mRNA of the infarction region. n = 3. *p < 0.05, **p < 0.01, ***p < 0.001. 0 represented the Ti_2_C-free cryogel ECP group, 4 represented the Ti_2_C-4-cryogel ECP group, and 8 represented the Ti_2_C-8-cryogel ECP group.

Angiogenesis is necessary for rescuing the survived cardiomyocytes to support myocardial repair 71. Herein, angiogenesis in the infarct region was assessed using microvessels (vWF) and arterioles (vWF and α-SMA) marker proteins by immunostaining. As shown in Figure 10, the round microvessels only expressed vWF proteins while the arterioles in circle shape co-expressed vWF and α-SMA proteins. The microvessels and the arterioles were significantly denser in the Ti_2_C-8-cryogel ECP transplantation group than those in the other groups. In the Ti_2_C-4-cryogel and the Ti_2_C-8-cryogel ECPs transplantation group, intact and well-defined arterioles structure could be easily observed, but few arterioles could be found in the Ti_2_C-free cryogel ECP transplantation group and the MI group. Furthermore, the gene expression of eNOS and VEGF remarkably higher in the infarction region in the Ti_2_C-8- cryogel ECP group than which in the MI group (Figure 10 C). In the repair process, M2 macrophage could recruit and promote the angiogenesis for wound healing. On the other hand, ECP with active CMs or stem cells was reported could promote the production of newly formed blood vessels and myocardial repair by paracrine effect 72, 73. In our study, The Ti_2_C-cryogel ECP derived microenvironment could enhance the implanted CMs maturation and functionalization so that could probably facilitate the paracrine of the CMs. On the other hand, the conductive Ti_2_C-cryogel also promoted vasculation *in vivo* via expression of the angiogenetic gene which could play an important role in nutrient delivery and cardiac function rebuilding in the infarction region. Taken together, these results suggested that Ti_2_C-cryogel ECP could be favorable for cardiomyocyte survival and promote angiogenesis, as a result, for repairing the damaged myocardium.

### The ECP and the Ti_2_C nanoparticles still located in heart four weeks posttransplantation

The degradation of transplanted ECPs and the distribution of the nanoparticles *in vivo* after transplantation is an important problem to discuss. Herein cy5 labeled Ti_2_C cryogel and Ti_2_C-free cryogel were synthesized for the implant tracing in rats. At day 28 after transplantation, fluorescence signals were only detected in the transplanted region of heart but not in other isolated organs (Figure S10). The high signal in the heart indicated that the transplanted scaffolds could exert its repair effects without obvious degradation in 4 weeks and kept their location in the transplanted region. Moreover, Ti_2_C distribution was analyzed by measuring the total concent of ti in various main organs. 208.67±30 μg/g and 210±41 μg/g of titanium were measured in the heart (attached with ECP) after transplantation for 2 days and 4 weeks respectively. Moreover, low titanium concent was detected in other off-target organs.

## Conclusions

Engineering cardiac patch (ECP) was a burgeoning treatment for myocardial infarction (MI) and the introduction of conductive materials was crucial for functional ECP construction. Here, the conductive MXene phase titanium carbide (Ti_2_C) nanoparticles were successfully synthesized by etching the MAX phase Ti_2_AlC and then were introduced into a dopamine-based cryogel to construct conductive Ti_2_C-cryogel. Just by simple sonication, Ti_2_C could be dispersed uniformly on the surface of the cryogel owning to electrostatic adsorption effect. The Ti_2_C-cryogel especially Ti_2_C-8-cryogel exhibited suitable elasticity and good conductivity, matching with those of the natural heart. Furthermore, owing to the synergistic effect of the biocompatible dopamine and conductive Ti_2_C, the developed Ti_2_C-cryogel could enhance cardiac cell retention and maturation. Typically, the intercalated discs with obvious desmosomes formed between the CMs seeded on Ti_2_C-8-cryogel. The fabricated Ti_2_C-8-cryogel ECP also exhibited overall and rhythmic intracellular Ca^2+^ puffs, along with synchronous contraction. After implanting the Ti_2_C-8-cryogel ECP onto the infarcted myocardium of MI rats for 4 weeks, the inflammatory reaction was remarkably suppressed, dense microvessels were triggered, and heart function was prominently improved. Consequently, the Ti_2_C-cryogel ECP could provide a suitable 3D microenvironment, facilitating the functional maturation of exogenous CMs and achieving a promising cardiac repair efficacy.

## Supplementary Material

Supplementary figures.

Supplemental movie 1.

Supplemental movie 2.

Supplemental movie 3.

Supplemental movie 4.

Supplemental movie 5.

## Abbreviations

DLS: dynamic light scattering
DOPA-MBA: dopamine-N′, N′-methylene-bisacrylamide
ECP: Engineered Cardiac Patch
EDX: energy-dispersive X-ray spectroscopy
EF: ejection fraction
GelMA: methylacrylic anhydride-Gelatin
LAD: left anterior descending
FS: shortening fraction
LVIDd: left ventricular internal diameter at end-diastole
LVIDs: left ventricular internal diameter at end-systole
MA: methylacrylic anhydride
MA-G: methacrylate-gelatin
MBA: dopamine-N'N'-methylene-bis-acrylamide
MI: Myocardial infarction
PEGDA: poly (ethylene glycol) diacrylate
RAECs: Rat aortic endothelial cells
SEM: Scanning Electron Microscopy
TEM: Transmission Electron Microscopy
Ti_2_C-free cryogel: cryogel without Ti_2_C nanoparticles
Ti_2_C-2-cryogel: Ti_2_C-cryogel with 2 mg/ml Ti_2_C final concentration
Ti_2_C-4-cryogel: Ti_2_C-cryogel with 4 mg/ml Ti_2_C final concentration
Ti_2_C-8-cryogel: Ti_2_C-cryogel with 8 mg/ml Ti_2_C final concentration
vWF: von Willebrand Factor
XRD: X-ray diffraction
