# Supplementary material for: Mussel-inspired conductive Ti2C-cryogel promotes functional maturation of cardiomyocytes and enhances repair of myocardial infarction
Source: Theranostics. 2020 Jan 12;10(5):2047–66. doi: 10.7150/thno.38876 (PMC7019164; doi:10.7150/thno.38876)
Supplement: Supplementary file 1 — Supplementary figures. [file thnov10p2047s1.pdf]

Mussel-inspired conductive Ti_2C-cryogel promotes functional maturation of cardiomyocytes and enhances repair of myocardial infarction

Genlan Ye[1,3#], Zubiao Wen[2#], Feng Wen[1], Xiaoping Song[1], Leyu Wang[1], Chuangkun Li[1], Yutong He[1,3], Sugandha Prakash[1], Xiaozhong Qiu[1*]

[1]Guangdong Provincial Key Laboratory of Construction and Detection in Tissue Engineering, School of Basic Medical Science; Biomaterials Research Center, School of Biomedical Engineering, Southern Medical University, Guangdong, Guangzhou 510515, China

[2]College of Chemistry and Chemical Engineering, Jiangxi Normal University, Nanchang 330022, China

[3]Guangzhou Regenerative Medicine and Health Guangdong Laboratory, Guangzhou 510005, China

[#] **These authors contributed equally to this work.**

[*]**Corresponding author:**

Prof. Xiaozhong Qiu

Guangdong Provincial Key Laboratory of Construction and Detection in Tissue Engineering, School of Basic Medical Science, Southern Medical University, Guangdong, Guangzhou 510515, China

Email address: qqiuxzh@163.com

Telephone number: 86-20-61648401

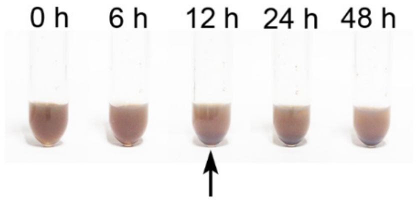

Figure S1. Graphene-cryogel prepolymer solutions rest for different times. Arrow showed aggregation occurred in 12 hours.

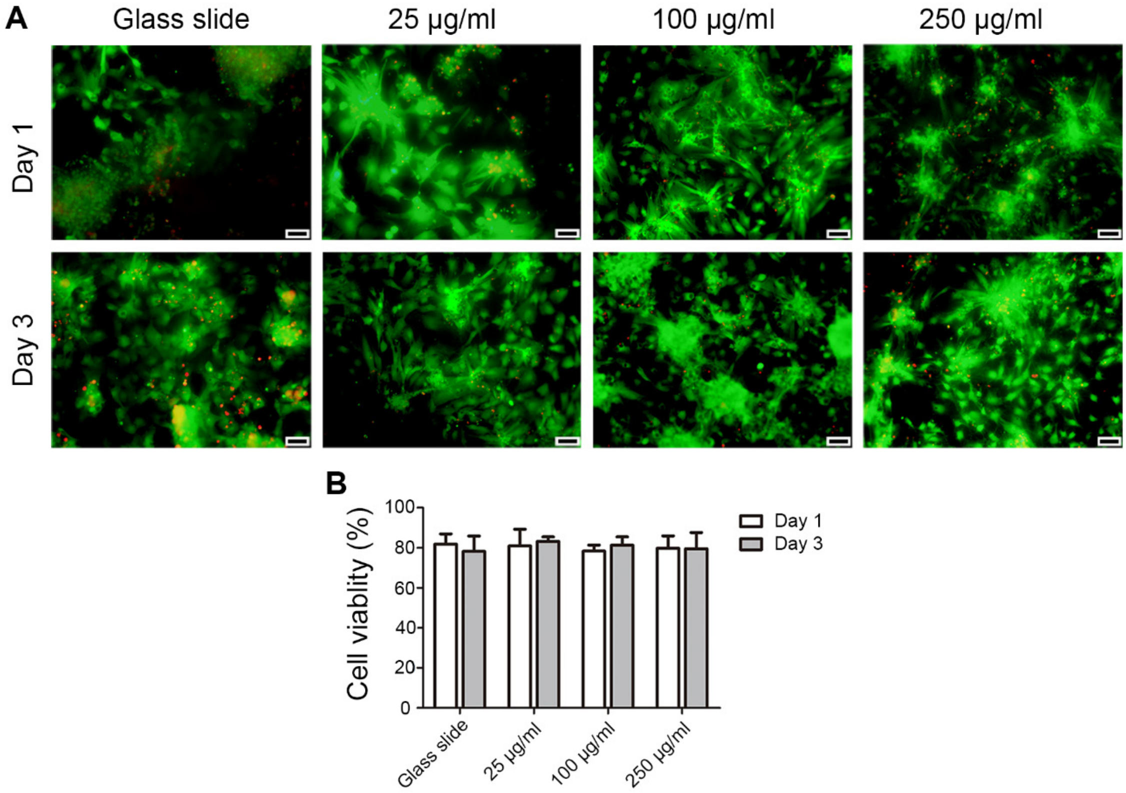

Figure S2. Biocompatibility of the MXene Ti_2C nanoparticles. A) Live-dead staining for neonatal rat CMs treated with various concentrations of Ti_2C nanoparticles on day 1 and day 3 of treatment. B) Quantitative cell viability of CMs based on the live-dead staining. Green cells represented live cells and red cells represented dead cells. Scale bars = 50 μm.

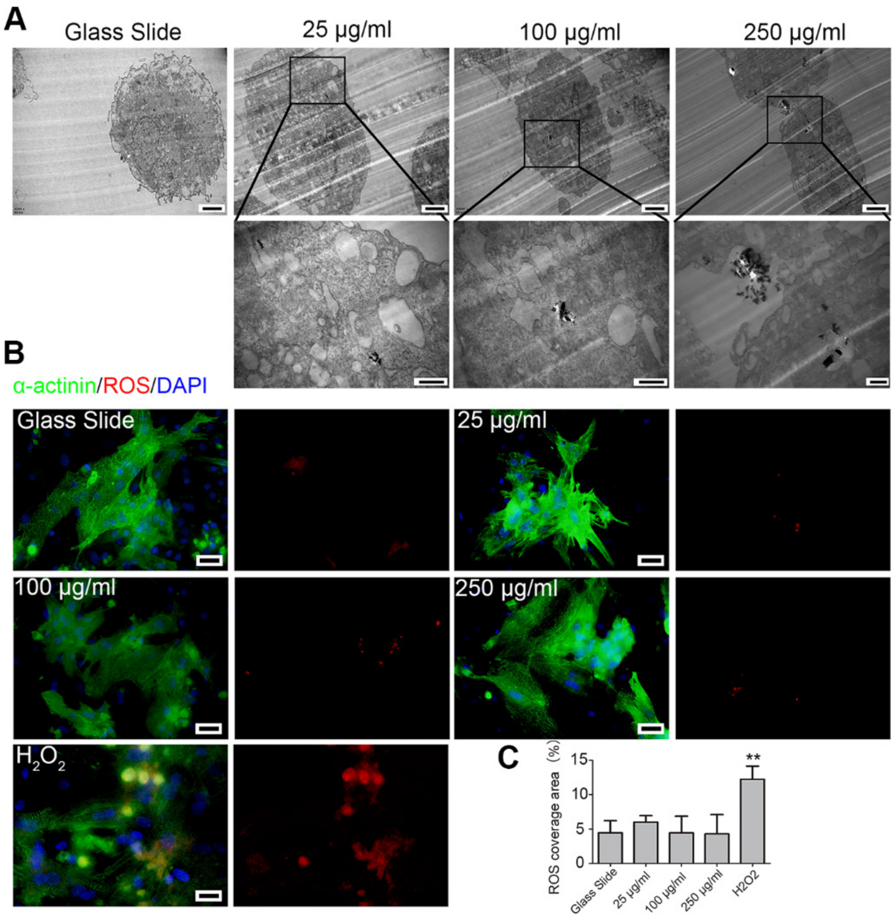

Figure S3. A few of Ti_2C nanoparticles invaginated into the CMs and did not elevate the cellular ROS levels. A) TEM of the CMs treated with diffident concentrations of Ti_2C nanoparticles. B) Fluorescence images of the ROS in CMs treated with a diffident concentration of Ti_2C nanoparticles. C) The quantification of the ROS level.

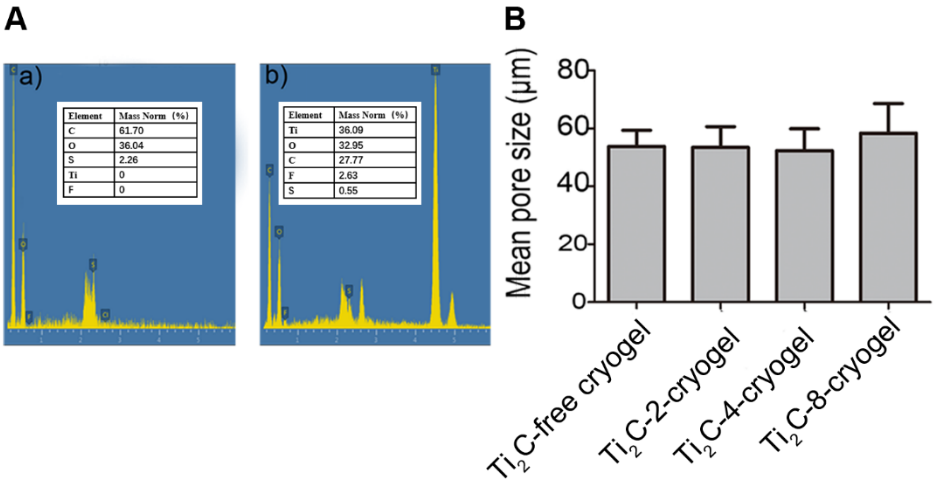

Figure S4. The characteristic of the Ti_2C cryogel. A) Element mass analysis of Ti_2C-free cryogel and Ti_2C-8-cryogel using energy-dispersive X-ray spectroscopy analysis. B) Mean pore sizes of different cryogels.

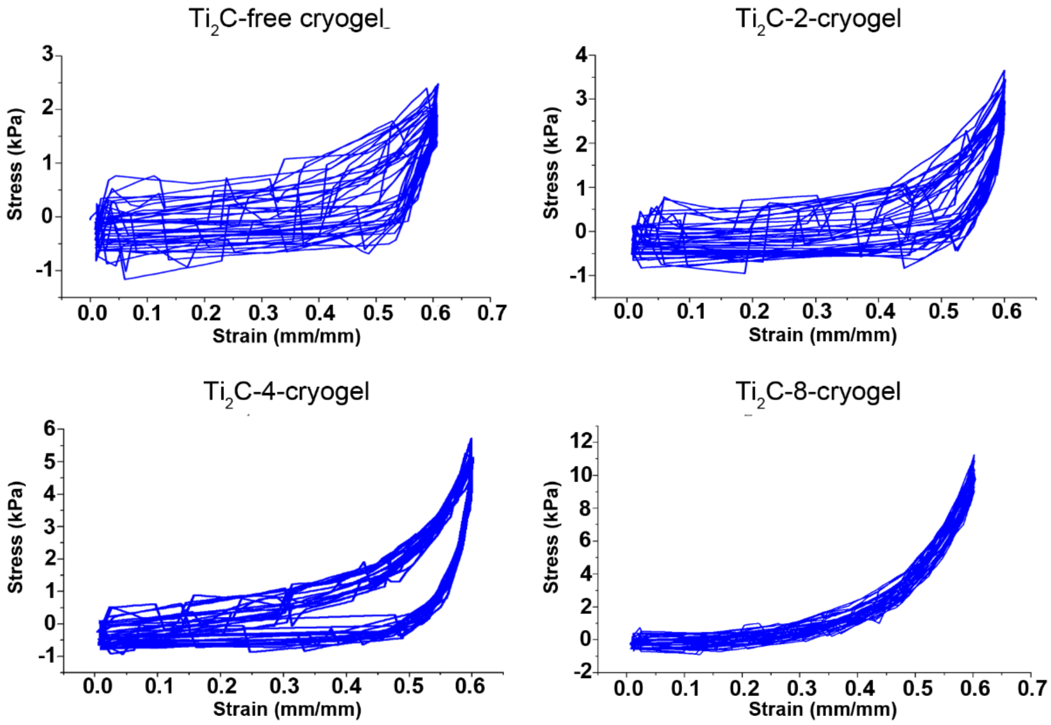

Figure S5. The stress-strain curve of different cryogels in compressive cycling test up to 60 % deformation for the first 20 cycles.

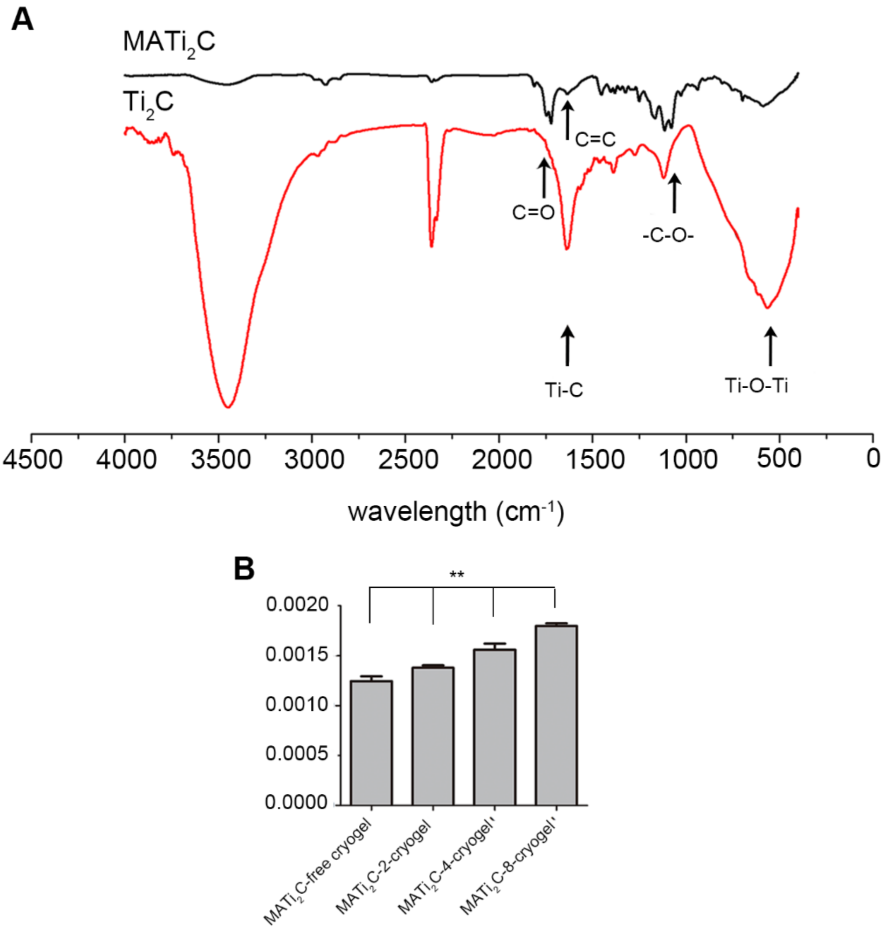

Figure S6. Characterization of $MATi_2C$ and $MATi_2C$-cryogel. A) FTIR spectrum of $MATi_2C$ and Ti_2C nanoparticles B) Conductivities of different $MATi_2C$-cryogels detected via four points probe method. n = 3. $^{**}p<0.01$

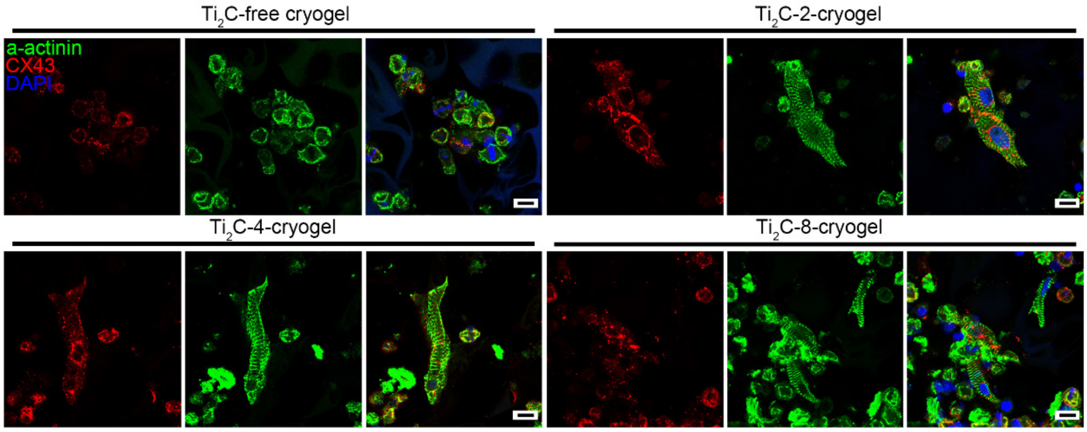

Figure S7. Immunofluorescent staining for cardiac-specific protein α-actinin (green) and CX43 (red) in CMs cultured on different Ti_2C-cryogels on day 3. Scale bars =10 μm.

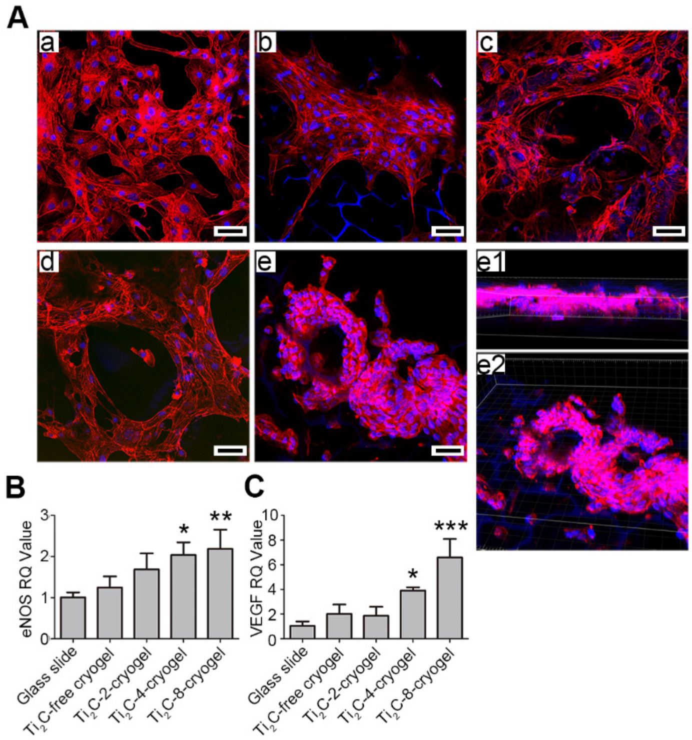

Figure S8. Ti_2C-cryogel promoted the tube formation of RAEC *in vitro*. A) F-actin showed the cell morphology of RAEC cultured on a glass slide or other different cryogels. 3D tube structure of RAEC formatted in Ti_2C-8-cryogels (a, glass slide, b, Ti_2C-free cryogel, c, Ti_2C-2-cryogel, d,Ti_2C-4-cryogel, e, Ti_2C-8-cryogel). B) and C) Expression of eNOS and VEGF genes in RAEC on a glass slide or other different cryogels. Scale bars=50 μm. n=3 *p<0.05, **p<0.01, ***p<0.001

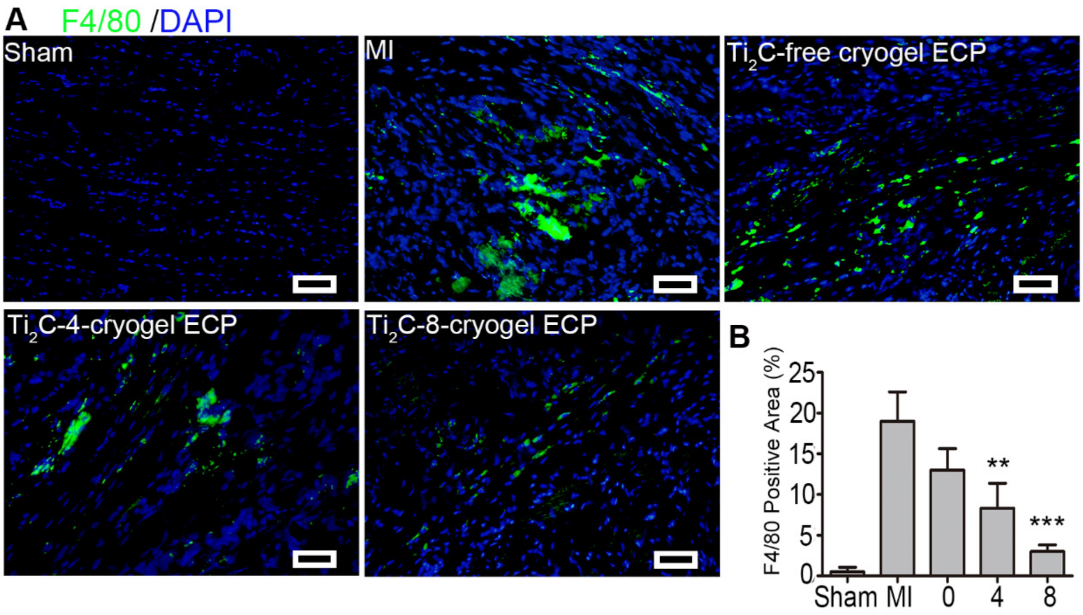

Figure S9. Inflammation of infarcted area after ECPs transplantation. A) F4/80 immunostaining in the infarcted area in different groups. B) F4/80-positive area in different groups. n = 3. $^{**}p<0.01$, $^{**}p<0.001$

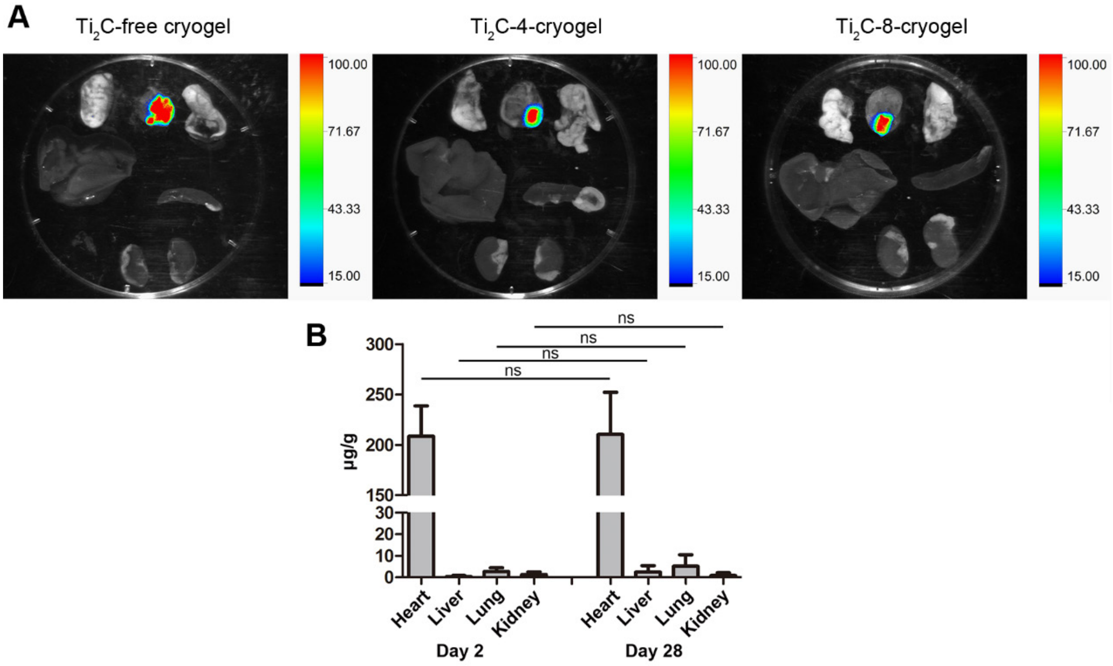

Figure S10. The location of the cryogel in rats and distribution of the Ti_2C nanoparticles in different organs in 28 days after transplantation. A) Fluorescence signals of cy5-labeled cryogels detected by a fluorescence imaging system. B) The total titanium content in different organs measured in 2 and 28 days after ECPs transplantation.

Supplemental movie 1. The compress and the release of the Ti_2C-8-cryogel;

Supplemental movie 2. The calcium transient in CMs cultured on different ECPs for 3 days;

Supplemental movie 3. The synchronous contraction of different ECPs *in vitro* on day 7 under the microscope;

Supplemental movie 4. The synchronous contraction of Ti_2C-8-cryogel ECP *in vitro* on day 7 observed by naked eyes.

Supplemental movie 5. The short-axis B model echo video of the ECP transplantation group.
